# Integrating a Combination HIV Prevention Intervention Into a Widely Used Geosocial App for Chinese Men Who Have Sex With Men: Protocol for a Single-Arm Pilot and Repeated Cross-Sectional Study

**DOI:** 10.2196/69536

**Published:** 2025-09-29

**Authors:** Wenting Huang, Kunru Ning, Shi Hao Ernest Koh, Guodong Mi, Fei Yu, Yufen Liu, Daniel Stegmueller, Kimberly A Powers, Stefan Baral, Patrick S Sullivan, Aaron J Siegler

**Affiliations:** 1 Department of Epidemiology Rollins School of Public Health Emory University Atlanta, GA United States; 2 Danlan Public Welfare Beijing China; 3 National Center for AIDS/STD Control and Prevention Chinese Center for Disease Control and Prevention Beijing China; 4 Department of Political Science Duke University Durham, NC United States; 5 Department of Epidemiology University of North Carolina at Chapel Hill Chapel Hill, NC United States; 6 Department of Epidemiology Bloomberg School of Public Health Johns Hopkins University Baltimore United States

**Keywords:** HIV, men who have sex with men, telemedicine, mHealth, protocol

## Abstract

**Background:**

HIV disproportionately affects men who have sex with men in China. However, HIV prevention services use among this population remains limited, in part due to concerns of privacy and sexual identity disclosure. These concerns might be addressed through telehealth services.

**Objective:**

This study was designed to assess the feasibility and acceptability of Blued+, which integrates an HIV prevention services package into a geosocial networking app commonly used by Chinese men who have sex with men.

**Methods:**

The study design was a single-arm pilot of the Blued+ intervention among men who have sex with men, using repeated cross-sectional surveys of an external population for comparison. For the pilot study, men were recruited from Beijing and Chengdu. A 3-month standard-of-care period with measurement at enrollment and month 0 (baseline) was followed by a 12-month intervention period with measurement at months 3, 6, 9, and 12. During the intervention period, participants received the enhanced version of the Blued app (Blued+) with HIV testing, linkage to care as needed, choice of condoms and condom-compatible lubricants, and pre-exposure prophylaxis (PrEP) services. PrEP was provided through in-app counseling and prescription, followed by lab tests at local clinics and mailed medication. Three cross-sectional surveys in months 0, 6, and 12 were administered to men in Beijing and Chengdu who were not enrolled in another HIV prevention study. These participants had access to the standard Blued geosocial networking app and local health services. The primary outcome of feasibility was the uptake of home HIV testing and PrEP. The coprimary outcome of intervention acceptability was measured with the System Usability Scale.

**Results:**

The run-in period was launched on July 15, 2022, with recruitment completed on August 24, 2022. The baseline period concluded in November 2022, and all follow-up assessments were completed by December 2023. At baseline, the pilot study enrolled 423 participants, and the cross-sectional comparison enrolled 1314 participants. Participants in the pilot were young (mean age 30, SD 7 years) and educated (324/423, 76.6% reported a college degree or higher), and most (302/423, 71.4%) reported HIV testing in the prior 3 months. Most participants (404/423, 95.5%) had heard of PrEP, and over a quarter had used PrEP (114/423, 27%). Participants in the comparison population had comparable sociodemographic characteristics, reporting HIV testing (501/857, 58.5%) in the prior 3 months, PrEP awareness (723/857, 84.4%), and PrEP use in the last 3 months (182/857, 15.4%).

**Conclusions:**

This pilot study will provide preliminary evidence regarding the feasibility and acceptability of the Blued+ intervention. The study findings will provide evidence setting the foundation for future research that involves embedding prevention platforms into apps that are already widely used. If preliminary impact is observed, future research would include a hybrid effectiveness implementation trial of the intervention.

**Trial Registration:**

ClinicalTrials.gov NCT06647173; https://clinicaltrials.gov/study/NCT06647173

**International Registered Report Identifier (IRRID):**

DERR1-10.2196/69536

## Introduction

Gay and bisexual men who have sex with men have been disproportionately affected by HIV in China [1,2], where the population of men who have sex with men was estimated to be approximately 21 million in 2018 [3]. HIV prevalence among men who have sex with men was estimated to have increased from 1% to 6% between 2001 and 2018 [4]. The estimated HIV incidence among men who have sex with men is much higher than for all other populations identified as being at risk of HIV in China since 2010 [5]. In Beijing, a cohort study of men who have sex with men documented 7.8% annual incidence [6]—a level similar to the highest incidence estimates among men who have sex with men subgroups in the United States [7]. In Chengdu, a southwestern city in China, the estimated incidence was 6/100 person-years between 2012 and 2018 [8] and 4.3% between 2018 and 2022 [9].

Despite the substantial epidemic, the use of HIV prevention services, including condoms, HIV testing, and HIV pre-exposure prophylaxis (PrEP), is low among Chinese men who have sex with men. In 2012-2016, fewer than half of men who have sex with men reported consistent condom use with male partners [10-12]. After a national intervention to expand HIV testing that started in 2007, the annual HIV testing rate increased from 21% to 38% by 2012, which was still suboptimal [13]. The suboptimal use of HIV prevention services has been associated with low self-perception of HIV risk [14] and HIV-related stigma [15,16]. To effectively control HIV transmission, a combination of multiple HIV prevention strategies, including biomedical (eg, PrEP use), behavioral (eg, HIV testing, condom, and lubricant use), and structural (eg, addressing stigma) approaches, is needed [17,18]. Therefore, adopting a combination HIV prevention approach that strategically and simultaneously leverages several types of interventions to address the specific needs of priority populations is prudent in that it is more likely to achieve comprehensive and sustained gains in HIV prevention [17].

Smart mobile devices have become ubiquitous in China and mobile apps have proliferated, offering an efficient approach to reach Chinese men who have sex with men with HIV prevention services [19]. Blued is an example of a men who have sex with men–focused app that provides dating and a wide range of HIV prevention and health information. Users can access these services anonymously and conveniently, regardless of their physical location [19]. Telehealth interventions using mobile apps have shown promising outcomes in improving practices such as consistent condom use and HIV testing to address disparities in sexually transmitted infections [20-24]. However, many innovative mHealth (mobile health) interventions that use apps have encountered challenges and issues in posttrial scale-up due to the lack of sustainable funding and limited technical expertise to support transitioning apps developed with research funding support into the marketplace [25,26]. Additionally, from the user perspective, having to manage and use many mobile apps for different purposes can result in information overload, which is associated with app use discontinuation [27]. To address these challenges, and in response to the National Institutes of Health’s call to develop and evaluate the feasibility and acceptability of combination HIV prevention interventions [17,18], we adopted a low-touch digital intervention approach in this pilot study, seeking to overcome future problems with scale-up by placing the prevention platform onto a pre-existing, widely used app.

This pilot study was developed on the Blued geosocial networking (GSN) app. Blued focuses on promoting a positive and healthy lifestyle for LGBTQ+ individuals. Launched in 2012 in China, it has gained considerable popularity both within China and globally, with 6 million active monthly users in China, including over 480,000 men who have sex with men in Beijing and 221,000 men who have sex with men in Chengdu (Guodong Mi, PhD, email communication, September 2018). Blued users can share their current precise locations to connect with and date users who are near them, match with others based on user interests, share photos and videos, and broadcast live streams. In addition to being a for-profit company, Blued was founded as a community organization and remains committed to public welfare. It collaborates with various community-based organizations to provide services in HIV prevention within the men who have sex with men community and advocates for antidiscrimination.

The aim of this pilot study was to assess the acceptability and feasibility of integrating a fully featured combination HIV prevention package into a widely used GSN mobile app. Our scientific premise is that the HIV prevention services continuum for men who have sex with men should be stronger and that integrating services into a mobile app already used by Chinese men who have sex with men will be feasible and acceptable, providing preliminary data to inform future assessment in a hybrid effectiveness implementation trial.

## Methods

### Study Design

**Figure 1:**
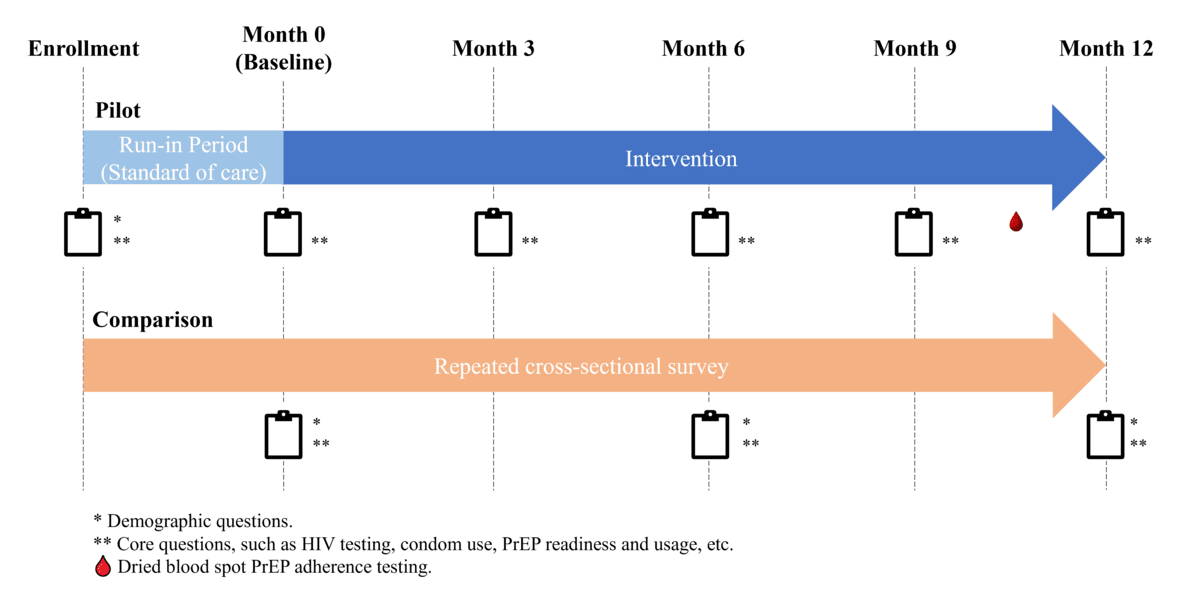
Study design overview of a study of HIV prevention service delivery to men who have sex with men through an HIV social networking app, China, 2022-2024. PrEP: pre-exposure prophylaxis.

This study was a single-arm pilot with a repeated cross-sectional design as comparisons (Figure 1). This research was funded as part of the National Institutes of Health Methods for Prevention Packages Program, which was focused on the development of combination HIV prevention interventions and therefore did not allow for randomized assessment [28]. To increase rigor in the absence of randomization, we included a 3-month run-in period and a series of repeated cross-sectional surveys in an external (comparison) population to assess pilot intervention performance. The set of three comparison groups over time allows the investigation of secular trends and potential impacts of panel attrition.

For the pilot study, a cohort of men who have sex with men was recruited from Beijing and Chengdu. Enrollment was followed by a 3-month standard-of-care (run-in) period, during which data on behaviors were collected but no HIV prevention content was provided, and a 12-month intervention period. Self-reported behavioral measures for the pilot study participants were collected at enrollment, baseline (month 0), and months 3, 6, 9, and 12. The set of three comparison groups was a series of cross-sectional surveys among men who have sex with men not enrolled in the intervention study, and administered at the same time intervals as the pilot study baseline (month 0) and months 6 and 12. Eligibility criteria for cross-sectional surveys were identical to those for the pilot study, with an additional exclusionary criterion of being enrolled in the intervention cohort. The set of three comparison groups was largely independent because they were advertised broadly to the whole city until the sample size of the study was obtained, although we did not exclude participation in subsequent comparison groups. For the intervention group, we also conducted an exploratory assessment of PrEP adherence among a subset of PrEP users willing to provide a specimen at month 12 follow-up with a blood-based biomarker test (detailed below).

### Recruitment

Participants for the pilot study and repeated cross-sectional surveys were recruited using the direct message function in the Blued app. Targeted inbox messages were sent to men who lived in the Beijing and Chengdu metropolitan areas. When interested men clicked on a link in the message, they were taken to a landing page containing basic information about the study. A brief screener survey then determined study eligibility.

### Eligibility

Eligible participants for both the pilot study and repeated cross-sectional surveys were registered Blued app users who self-reported male sex at birth, male gender identity, age ≥18 years, having had anal sex with a man in the last 6 months, having HIV-negative or unknown HIV status, residing in Beijing or Chengdu, not enrolled in another HIV prevention study, and being eligible for PrEP according to China’s consensus statement [29]. According to the consensus statement, men who have sex with men are eligible if they ever used PrEP or postexposure prophylaxis (PEP) before or are willing to use PrEP or PEP, or meet at least one of the following four criteria in the past 6 months: (1) engaged in any condomless anal or vaginal sex, (2) injected drugs or shared needles, (3) had a serodifferent partner known to be living with HIV, or (4) were diagnosed with a sexually transmitted disease [29].

### Enrollment

The advertisement and enrollment process for the pilot study and repeated cross-sectional surveys were similar. Study advertisements were sent as direct messages to Blued users in Beijing and Chengdu based on their log-in geolocations in the prior 3 months. Preliminarily eligible participants were asked to provide their contact information and complete the consent process. Given the remote nature of consent, we verified understanding of the key elements of the consent form with a brief quiz. Correct responses and brief explanations of key components in the consent form were shown when participants selected an incorrect option. Upon successful completion of the consent quiz, participants indicated consent by electronically affirming their decision to participate in the study.

### Intervention Development

To inform intervention design, we obtained information about app preferences and user experience with the existing app, Blued. We conducted 3 in-person activity-based focus group discussions (FGDs) in Beijing with a range of 6-10 Blued users in each group in April-May 2021. The eligibility criteria were the same as the criteria above for the pilot study and the repeated cross-sectional survey. The length of Blued use ranged from 2 to 9 years. During the FGD, participants were first asked to review the HeHealth portal in the existing Blued app, including the home, condoms or lubricants, and HIV testing kits pages. Then, participants were provided with visual illustrations of concepts regarding how PrEP care components could be incorporated into the Blued+ app, including PrEP education information, PrEP screening quizzes, no-cost PrEP care and referrals, reminders, and peer communication. The FGD guide can be found in Multimedia Appendix 1. A rapid qualitative analysis of the FGDs was conducted to summarize participants' feedback on each function to inform Blued+ app development.

In general, FGD participants expressed a preference for a simple and easy-to-navigate page design. Regarding the HIV testing kits page, participants suggested a graphic display mode to present different types of test kits clearly. For condom and lubricant products, participants preferred thin condoms with good quality from reputable brands. Regarding PrEP care services to be added in Blued+, participants suggested that the page should be tailored in terms of PrEP-related information. For PrEP screening quizzes, participants were concerned about the lack of explanation about the results and emphasized the need to clarify the purpose of the quiz beyond simply encouraging people to purchase PrEP medication. To access PrEP care, most participants preferred to talk to a doctor to obtain PrEP prescriptions and medication, but they also worried about potential discrimination and stigma attitudes at in-person clinics. When considering PrEP care support services like reminders and peer communication, participants suggested that the reminder service could be offered as an optional service, but that peer communication would be unnecessary. To address concerns about PrEP efficacy, side effects, and access to prescriptions and medication, participants suggested the need for an in-app clinician for consultation, which we added to the intervention.

### Standard of Care

During the 3-month standard-of-care period in the pilot study and for the cross-sectional comparison assessments, participants were able to access the existing Blued app (ie, without the additional features described above). Blued began as a community-based organization and maintains a strong community presence. This includes a network of HIV testing locations and a series of HIV testing promotion ads in Blued. In 2020, Blued started to offer a set of digital sexual health services in a for-profit model, HeHealth. It provides users with access to mailed home HIV tests, mailed standard condoms, and water-based lubricants for purchase, all in a manner similar to retail pharmacy services. HeHealth also offers pharmacy PrEP services, with users who already have a PrEP prescription from their local clinic able to receive PrEP through package delivery.

### Intervention

#### Overview

During the intervention period, pilot study participants received the enhanced version of the Blued app: Blued+. The installation was integrated with a regular Blued app update to avoid disrupting users. A notification about the update was sent to study participants. Once study participants agreed to update the Blued app on their smartphone, the update to the enhanced Blued+ version was automatic.

The theoretical foundation that structured the integration of the HIV prevention services into this app environment was the Extended, Unified Theory of Acceptance and Use of Technology (UTAUT2). UTAUT2 is a frequently used model for the development and assessment of technology-based health interventions, with over 20,000 citations and recent validation for mobile technology assessment in China [30,31]. Domains relevant to Blued+ were performance expectancy (PE), effort expectancy (EE), social influence (SI), and habit (H) (Table 1). Blued+ sought to provide users services in a manner they deem valuable (PE), to decrease expected effort expended by using functions and design layout consistent with the existing Blued app (EE), to include health-positive messages encouraging self-care in direct messaging for social influence (SI), and to use reminder systems to help users build HIV prevention habits (H).

**Figure 2:**
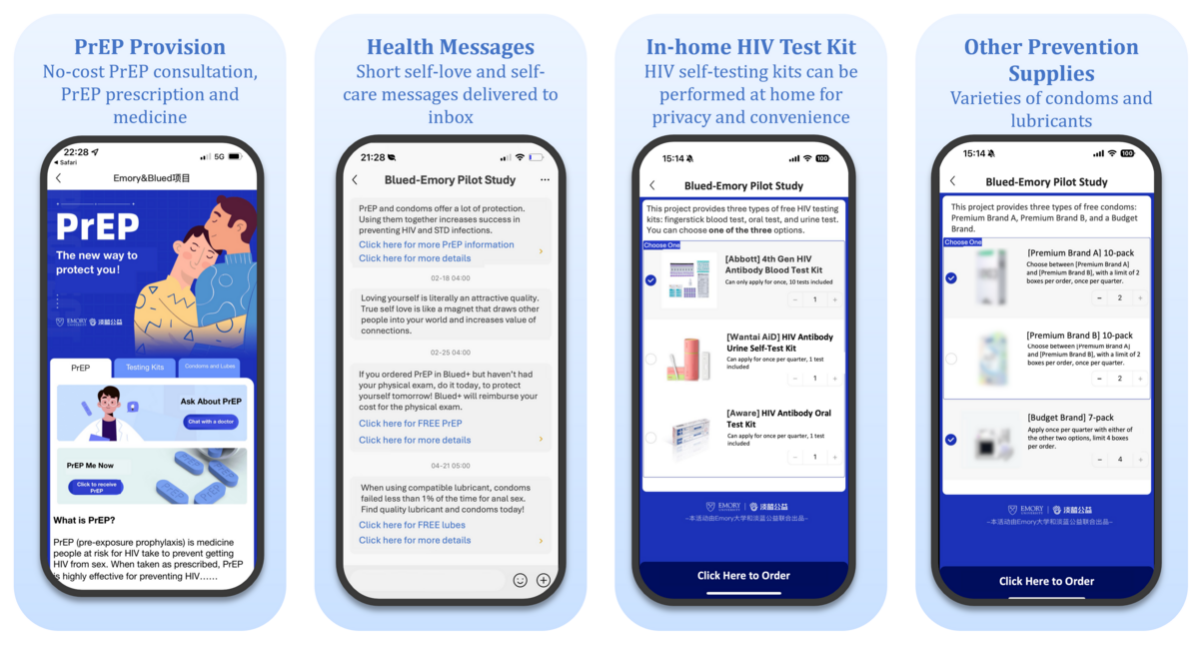
Screenshots of interventions in the Blued+ app. PrEP: pre-exposure prophylaxis.

“Video walk-throughs” of telehealth intervention apps have been previously suggested [25]. In Multimedia Appendix 2, we provided a video walk-through of the Blued+ app to facilitate a detailed understanding of how intervention features were offered to users. Screenshots of some pages from the Blued+ app, translated into English, were presented in Figure 2. Services in the HIV prevention intervention package included PrEP provision, in-home HIV tests and linkage to care, and prevention supplies such as condoms and lubricants. Additionally, participants received weekly health messages focused on PrEP, sexual health, and self-care. All these services were provided at no cost.

**Table 1:** Theoretical framework, domains, and intervention design.

UTAUT2^a^ constructs	Definition	Intervention design
Performance expectancy	The degree to which an individual believes that using the system will help him or her to attain gains in health.	Provide in-home HIV testing kit, condoms, lubricant, and PrEP^b^ at no cost.
Effort expectancy	The degree of ease is associated with the use of the system.	Use functions and design layout consistent with the existing Blued app.
Social influence	The degree to which using the new system is appreciated in the social network that is important to the individual.	Send highly rated health-positive messages encouraging self-care.
Habit	The extent to which people tend to perform behavior automatically because of learning.	Use health messaging to help build habits of using HIV prevention services on the enhanced Blued app.

^a^UTAUT2: Extended, Unified Theory of Acceptance and Use of Technology.

^b^PrEP: pre-exposure prophylaxis.

#### Health Messages

Health messages were adapted from a set of messages used in an app-based messaging and services intervention (M-cubed study) that was found to increase HIV testing and PrEP use among men in the United States [32]. The messages were initially translated into Chinese by staff in the research team, then reviewed and revised by staff at Blued to ensure their cultural and contextual appropriateness for Chinese men who have sex with men. Health messages in the study were sent at intervals of 1-3 weeks with a total of 29 messages sent out to each participant.

Topics included promotion of PrEP use (n=11), condom and lubricant use (n=6), HIV testing (n=6), engagement in health care (n=2), and health-positive messages encouraging self-care (n=3). PrEP use support messages included information on PrEP and PrEP medication, PrEP effectiveness, PrEP costs, side effects, dealing with PrEP stigma, and how to access PrEP. Condom use messages included traditional messages encouraging consistent condom use and encouraging condom use with lubricants for pleasure. HIV testing messages included information about rapid testing, where to get tested, and encouraging regular testing every 6 months. Messages about engagement in health care encouraged users to talk to their health care providers and offered the in-app clinician counseling option. Positive self-support messages were adapted from a set of self-care and self-love messages provided by a community PrEP educator who focused on mental well-being and harm reduction behaviors. For example, one of the self-love messages was “To love oneself is the beginning of a lifelong romance” [Oscar Wilde]. A team-based translation approach was used to translate these messages into Chinese. Two bilingual staff from the research team and two bilingual local staff members worked together to ensure the messages were accurate and culturally appropriate. For most messages, a hyperlink with an appropriate destination was included within the message. For instance, a hyperlink within a PrEP use message “Could PrEP be right for you? Chat with our Blued+ provider” would direct users to the page where they could consult with a PrEP provider. A hyperlink within the message “Got a hot date? Don’t go unprepared. Get some free condoms and lube today!” would direct users to the page to order condoms and lubricants. A full version of all health messages with English translations can be found in Multimedia Appendix 3.

#### PrEP Care

The clinical eligibility for PrEP use in China are (1) laboratory-confirmed HIV-negative, (2) no contraindications to emtricitabine or tenofovir disoproxil fumarate, (3) creatinine clearance of at least 60 mL/min (reported by a laboratory), (4) willingness to adhere to a PrEP regimen, and (5) willingness to attend PrEP maintenance visits every 3 months.

Participants interested in accessing PrEP could click on the link in PrEP use messages or access services directly in the Blued+ app that schedules a PrEP counseling session with an in-app clinician. After counseling, participants who decided to initiate PrEP could get lab tests for HIV, syphilis, hepatitis B surface antigen (HBsAg), and serum creatinine from any local tier-2 or tier-3 hospital. The age and weight of the participant were collected by self-report to calculate the creatinine clearance rate. Prior to the study launch, 8 staff from Blued (4 in each city) went to a hospital of their choice and got lab tests to trial the process and estimate its cost. The cost associated with completing these lab tests was estimated to range from US $27 (190 CNY) to US $50 (350 CNY). Reimbursement for tests was sent once participants uploaded the test results to Blued+ in the app. The test results were reviewed by a study clinician, and participants were prescribed PrEP in the platform if they were eligible for PrEP. After the prescription, research staff received a notification and shipped the medication within 1 week of an order, in batches of 90 pills to cover daily PrEP use for 3 months. Participants received tracking numbers once the medicine was shipped. Participants could refill every 3 months if they received appropriate laboratory testing specified in the Chinese guidelines [29]. PrEP medicines used in this study were pills containing 200 mg emtricitabine and 300 mg tenofovir disoproxil fumarate.

#### In-Home HIV Test and Linkage to Care

In Blued+, participants could order in-home HIV tests by clicking a link in HIV testing health messages or clicking a tab in the Blued+ app. The ordering page was designed to be easy to navigate to reduce users’ effort expectancy. Similar to the procedures for shipping PrEP medication, once the order was placed, the Blued+ pharmacy received a notification and prepared the shipment. Three types of in-home HIV test kits, using capillary blood, urine, or oral swabs, were offered to maximize participant choice. During the intervention period, each participant could order a package of 10 blood test kits, 1 urine test kit, or 1 oral test kit. The aim of offering 10 blood test kits (anticipated to be the most frequently selected option) was to provide enough test kits for the 12-month intervention period, allowing participants to test as frequently as they would like. Participants could upload photos of their test results to the app and receive free counseling (regarding the test kit and result interpretation) and linkage to care when appropriate from a study coordinator at Blued. A free message app account number of the in-app clinician was also provided to participants so that they could reach out for HIV test counseling and other related questions. Participants were able to use the Blued+ platform to voluntarily upload their HIV self-test results for further counseling. Experienced counselors would provide further explanation of the test result and referral to clinics for confirmatory tests, when necessary, through phone calls.

#### Other Prevention Supplies (Condoms and Lubricants)

Three types of condoms with different thicknesses and brands (ultrathin vs standard and premium vs budget brand) and water-based and silicone-based condom-compatible lubricants were available for participants to order from the platform. The types of condoms and lubricants were selected based on our target users’ preferences as assessed during FGDs (see above). The ordering process for condoms and lubricants was similar to ordering HIV testing kits. When participants added condoms to their “cart” in Blued+, a note would pop up reminding them to add lubricants to use with condoms.

For condoms, each participant was offered 48 condoms per quarter. This number was based on 2-3 sexual activities per week, a common sex frequency for men who have sex with men in China [33]. The Blued+ platform allowed participants to choose luxury condoms of either premium brand A (ultrathin 20 condoms) or premium brand B (air-feel 20 condoms). This gave participants the opportunity to try different brands and thicknesses. Additionally, each participant could order 28 standard condoms from a budget brand.

It is recommended that 5-10 mL of condom-compatible lubricant be used with each anal sexual activity [34,35]. Participants were offered a total of 340 mL of lubricants per quarter, which included 1 container of water-based lubricant (100 mL) and 2 containers of silicone-based lubricant (120 mL each).

#### Differences Between Standard of Care and Intervention

Although the standard of care has some similarities to the intervention, there were notable differences informed in part by the study’s qualitative assessments. The health page in the intervention was designed to be simpler and easier to navigate. Health messages in the intervention covered a wide range of HIV prevention–related topics and health-positive self-care messages, whereas the health messages in the standard of care only covered a narrower range of HIV prevention topics such as condom use and HIV testing, and there was no systematic delivery of these messages. For HIV prevention products, the health portal in Blued+ offered options of different condoms, lubricants, and in-home HIV test kits based on user preferences. For PrEP care access, the intervention offered in-app PrEP counseling and prescribing, and medication mailing when appropriate. All these services were provided at no cost to participants in the pilot.

### Data Collection

#### Pilot Cohort Survey and Cross-Sectional Survey

Data were collected through Sojump, a widely used and secure online survey platform in China that offers survey design features including multiple question types and branching logic. The survey link was sent to participants through the direct message function in the Blued app. Blued user IDs, tied to unique phone numbers, were used to identify and exclude duplicate responses within the cohort survey as well as between the cohort survey and the repeated cross-sectional survey.

The enrollment survey included questions in the domains of (1) sociodemographic characteristics, (2) relationship status, (3) sexual history, (4) HIV and STI testing history, (5) PrEP knowledge or attitudes, (6) PrEP intention and use (including adherence), (7) HIV knowledge, (8) PrEP stigma, (9) mental health, and (10) prevention supplies order history. Sociodemographic characteristics questions were adapted from the annual American Men's Internet Survey (AMIS) [36]. HIV knowledge was assessed using an 8-item scale from the Sustainable Health Center Implementation PrEP Pilot (SHIPP) study [37]. PrEP stigma was measured using a previously validated scale [38]. Mental health was measured using the Patient Health Questionnaire for Depression and Anxiety (PHQ-4) [39]. The full enrollment survey can be found in Multimedia Appendix 4. Follow-up surveys include the domains of (1) relationship status, (2) sexual history, (3) HIV and STI testing history, (4) PrEP knowledge or attitudes, (5) PrEP intention and use, (6) prevention supplies order history, and (7) acceptability of Blued+. HIV knowledge, PrEP stigma, and mental health were only included in the month 6 and month 12 follow-up surveys because they were believed to be less labile. Acceptability of Blued+ was measured by the widely used System Usability Scale that assesses the subjective usability of products and systems [40,41]. The acceptability questions were only included in the month 12 follow-up survey. Questions in the pilot cohort survey and cross-sectional survey were identical. The average survey completion time ranged from 10 to 20 minutes across participants at each time point.

#### Dried Blood Spot

In addition to self-report data regarding PrEP adherence collected from the cohort survey, we conducted an exploratory assessment of the suitability and acceptability of biomarker data collection to measure drug levels in blood samples. Participants who initiated PrEP were asked at an interim visit between months 9 and 12 to visit a local laboratory for the collection of a blood specimen to create a dried blood spot to measure tenofovir diphosphate (TFV-DP) levels in plasma. For this procedure, participants were first asked to complete a supplemental survey and to receive an HIV screening test. The survey assessed PrEP pill usage in greater detail than in the cohort survey to evaluate adherence to their regimen of either daily use or on-demand use in the past 2 months. A trained phlebotomist at the designated local laboratory then collected finger-prick blood onto a paper card to create a dried blood spot card for future testing for TFV-DP. For daily pill use, TFV-DP levels provide an indicator of the mean number of days per week that PrEP is ingested over a period of approximately 1-2 months [42]. For daily users, a cut point of >700 fmol/punch TFV-DP indicates >4 doses/week and thus serves as a surrogate for substantial protection from HIV infection. For event-based PrEP users, adherence was determined by evaluating if the TFV-DP level matches with the self-report behavior data on sexual frequency.

### Statistical Analysis Plan

#### Primary Analysis

The primary analysis will be focused on the feasibility and acceptability of the pilot intervention, assessed based on a series of benchmarks (Table 2); these benchmarks were established in 2018 when the research was first proposed. The intervention will be determined as feasible if a minimum proportion of participants receive services for home HIV testing (30%) and PrEP prescription (20%) at month 12. Minimum targets were determined based on modeling that indicate levels of service uptake required to substantially influence an HIV epidemic [43]. The intervention will be considered acceptable if it reaches a System Usability Scale rating benchmark score of “good” (≥71 out of 100). Analyses will be conducted using SAS 9.4 (SAS Institute) or similar statistical software packages.

**Table 2:** Study measures, sources of data, and definitions for primary and secondary outcomes for a pilot study of integration of biomedical prevention services in an existing social networking app for Chinese men who have sex with men, Beijing and Chengdu, 2022.

Construct			Measure		Data source		Definition		Analysis
**Primary outcomes**									
	Feasibility	Home HIV test orders		Order fulfillment		Proportion of participants sent a home HIV test		≥30%	
	Feasibility	PrEP^a^ initiation		Clinical site data		Proportion of participants receiving a PrEP prescription		≥20%	
	Acceptability	System Usability Scale [40,41]		Survey		10-item scale, good score is ≥71		Good score (≥71 out of 100)	
**Secondary outcomes (preliminary efficacy assessment)**									
	HIV testing	HIV testing		Survey		Proportion of participants reporting HIV testing in the last 3 months		Descriptive, longitudinal analysis^b^	
	Condom use	Condom use		Survey		Proportion of participants reporting always using condoms for anal sex in the last 3 months		Descriptive, longitudinal analysis^b^	
	PrEP use	PrEP use [44]		Survey		Proportion of participants reported PrEP use in the last 3 months		Descriptive, longitudinal analysis^b^	

^a^PrEP: pre-exposure prophylaxis.

^b^Descriptive analysis: outcome proportions over time; longitudinal analysis with generalized estimating equations or similar models to measure predictors of outcomes.

#### Statistical Power and Sample Size

This is a pilot study assessing feasibility and acceptability; therefore, our sample was not powered to detect significant differences in the intervention period compared to the standard-of-care period (before baseline) or to the set of three cross-sectional comparisons. A sample of 200 pilot study participants per intervention city was selected based on budget and time constraints. This sample size was also anticipated to provide estimates of key parameters with sufficient precision. For instance, a 95% CI for the prevalence of our binary benchmarks (home HIV test orders, condom package orders, PrEP medication orders) would have a half-width of 5.1 percentage points. This is calculated assuming an estimated population SD of 0.5 and Pr(w)=0.9 [45], where Pr(w) represents the probability that the width of a future CI will not exceed 95% and accounts for the fact that the SD is not known and will therefore lead to CIs that vary from sample to sample [46].

#### Additional Analysis

Additional analysis will include assessing preliminary efficacy with descriptive endpoints and measuring changes from baseline to intervention periods in HIV testing, condom use, and PrEP use. Descriptive analyses will include comparisons of the proportions of users reporting use of condoms, PrEP, and HIV testing in the pilot relative to the set of three comparison groups (Table 2). Longitudinal analyses will assess trends within the pilot cohort while accounting for autocorrelation, using models such as generalized estimating equations to measure predictors of the binary outcome of HIV testing, condom use, and PrEP uptake. Models will contain the 7 time periods with primary comparisons of 0-month (first baseline) measurement to 6-month (end wash-out baseline period) measurement and to 12-month (end intervention period) measurement. Time will be considered as a fixed effect to compare proportions tested across intervals, with a random intercept for individuals. TFV-DP data will be reported descriptively to provide context for the self-report PrEP use data.

### Ethical Considerations

The study was approved by the Emory institutional review board (IRB00116983) and the Blued institutional review board in January 2020. Study participants completed the consent process, as described above in the Enrollment subsection, before any study activity started. Participants had the right to withdraw from this study at any time without any penalty.

A role-based access control was applied to the database storage. Participants’ identifying information (eg, contact information such as Blued user IDs and phone numbers) can only be accessed by the study coordinator in China to identify and exclude duplicate responses to the cohort and cross-sectional surveys. Once the deduplication process was complete, a nonpersonally identifying study ID was used to store all study data (deidentified). Data received by other study team members, including all collaborators in the United States, were deidentified.

A compensation of US $15 (100 CNY) was provided for each of the cohort surveys and repeated cross-sectional surveys. An additional US $60 (400 CNY) was provided for each participant who completed all 6 cohort surveys in the pilot study. For each participant who completed the dried blood spot collection, an additional US $45 (300 CNY) compensation was provided.

## Results

The enrollment was launched in July-August 2022. The baseline was concluded in November 2022. All follow-up assessments were completed in December 2023. As part of recruitment efforts, the study team sent out direct messages to individual app users in the study cities. The screening survey link was opened 6445 times. A total of 625 participants were screened as eligible after deduplication. Based on the prior determined sample size, 440 participants were contacted to be enrolled in the pilot study. After the 3-month run-in period, 423 were retained to start receiving the intervention. Retention rates at 3, 6, 9, and 12 months were 96.2% (407/423), 94.6% (400/423), 94.3% (399/423), and 94.3% (399/423). For the cross-sectional comparison, the baseline cross-sectional survey was opened 6565 times, and 857 participants were eligible after deduplication. The number of participants recruited for the 6-month and 12-month cross-sectional surveys were 764 and 747, respectively.

Sociodemographic and behavioral characteristics at baseline for each group are presented in Table 3. Participants enrolled in the pilot had an average age of 30 (SD 7.4) years. Slightly more pilot participants resided in Beijing than in Chengdu. More than two-thirds of the pilot participants had obtained at least a college degree, and about three-quarters reported working full-time. Over a third of participants had a monthly income of over US $1400, a level that is above the average income level of urban nonprivate position employees. Most participants self-identified as homosexual. In terms of behaviors, over half of the participants (265/423, 62.6%) reported having condomless anal sex in the prior 3 months. About two-thirds (302/423, 71.4%) of participants had been tested for HIV in the prior 3 months. PrEP awareness was high—nearly all participants (404/423, 95.5%) had heard of PrEP, among whom 27% (114/423) had used PrEP. Of the 114 participants who had used PrEP, two-thirds (80/114, 70.2%) had used PrEP in the prior 3 months.

Participants in the cross-sectional comparison group had similar sociodemographic characteristics to those in the pilot study, except the comparison group had a slightly higher proportion of participants residing in Beijing (565/857, 65.9%) compared to the pilot study participants (230/423, 54.4%; *P*<.001). Lower proportions of participants in the comparison group reported testing for HIV in the prior 3 months (501/857, 58.5%; *P*<.001), compared to the pilot study population. Lower proportions of participants in the comparison group also reported PrEP awareness (723/857 [84.4%] vs 404/423 [95.5%]; *P*<.001), ever PrEP use (182/857 [21.2%] vs 114/423 [27%]; *P*=.02), and PrEP use in the prior 3 months (132/857 [15.4%] vs 80/423 [18.9%]; *P*=.11).

**Table 3:** Sociodemographic and behavioral characteristics of study participants in the pilot and comparison groups at baseline, Beijing and Chengdu, 2022.

		Pilot (n=423)	Comparison (n=857)	*P* value^a^
Age (years), mean (SD)		30 (7.4)	30 (7.8)	>.99
**City, n (%)**				<.001^b^
	Beijing	230 (54.4)	565 (65.9)	
	Chengdu	193 (45.6)	292 (34.1)	
**Education, n (%)**				.06
	Postgraduate and above	89 (21)	216 (25.7)	
	College or university	235 (55.6)	455 (53.1)	
	Associate’s degree or technical school	84 (19.9)	137 (16)	
	High school and below	15 (3.5)	49 (5.7)	
**Employment status, n (%)**				.08
	Employed full-time	328 (77.5)	675 (78.8)	
	Employed part-time	31 (7.3)	38 (4.4)	
	Full-time student	43 (10.2)	95 (11.1)	
	Part-time student	2 (0.5)	2 (0.2)	
	Unemployed or retired	13 (3.1)	42 (4.9)	
	Others	6 (1.4)	5 (0.6)	
**Income (monthly, in US $), n (%)**				.70
	>1400	173 (40.9)	365 (42.6)	
	980-1400	96 (22.7)	205 (23.9)	
	420-979	95 (22.5)	185 (21.6)	
	<420	59 (13.9)	102 (11.9)	
**Sexual orientation, n (%)**				.26
	Homosexual	357 (84.4)	696 (81.2)	
	Bisexual	65 (15.4)	155 (18.1)	
	Heterosexual and others	1 (0.2)	6 (0.7)	
**Condomless anal sex, past 3 months, n (%)**				.2
	Yes	265 (62.6)	505 (58.9)	
	No	158 (37.4)	352 (41.1)	
**HIV testing, past 3 months, n (%)**				<.001^b^
	Yes	302 (71.4)	501 (58.5)	
	No	121 (28.6)	356 (41.5)	
**Heard of PrEP^c^, n (%)**				<.001^b^
	Yes	404 (95.5)	723 (84.4)	
	No	19 (4.5)	134 (15.6)	
**PrEP use, ever, n (%)**				.02
	Yes	114 (27)	182 (21.2)	
	No	309 (73)	675 (78.8)	
**PrEP use, past 3 months, n (%)**				.11
	Yes	80 (18.9)	132 (15.4)	
	No	343 (81.1)	725 (84.6)	

^a^*t* test for age; Fisher exact test for the rest.

^b^*P*<.005, below Bonferroni-adjusted critical value.

^c^PrEP: pre-exposure prophylaxis.

## Discussion

### Importance of the Study

This protocol describes a pilot study assessing the feasibility and acceptability of an mHealth intervention that integrated HIV services into an existing GSN mobile app for men who have sex with men in China. A number of previous telehealth studies have developed study-specific mobile phone apps to deliver interventions [20,47,48]. Although these interventions are frequently effective in changing behaviors, few of them maintained changes over time [23]. Challenges of “translation” and obtaining “downloads” are the primary barriers to bringing the app from research into practice and maintaining the app [49]. Integrating interventions into a widely used app may overcome these challenges. Translation is a common implementation challenge. Effective health apps developed by researchers may never be translated into a sustainable model for public and long-term use [25,26]. Translating a study app into a marketable business product with sufficient profit to sustain the intervention is often out of the scope of research studies and out of the capability of researchers, who frequently lack expertise and financing to accomplish this critical task [22-24]. The challenge of downloads is that once the app is translated into the market, convincing eligible individuals to download the app may be challenging unless they are aware of the app and see a clear benefit of having it [49]. Given these barriers, we offered HIV prevention services in an app that already has a sustainable model and is widely downloaded by Chinese men who have sex with men, rather than developing a new app. To our knowledge, this is the first study incorporating interventions into a widely used GSN app for men who have sex with men. This study’s results may inform future efforts to optimally leverage mHealth resources by determining the feasibility and acceptability of building prevention models into existing apps.

Stigma related to sexual orientation and HIV has been identified in other studies as a barrier for Chinese men who have sex with men accessing health care services, particularly HIV prevention services [50,51]. Moreover, prior studies found that higher levels of stigma were associated with lower awareness and use of PrEP [52,53]. In addition to sexual orientation and HIV-related stigma, stigmatization of PrEP use can be a barrier for PrEP initiation and adherence [54,55]. Therefore, our study measures possible change in HIV and PrEP stigma that could occur with the use of tailored services, although reducing stigma was not the primary intention or outcome of this pilot study.

Participants in the pilot and comparison groups were similar at baseline with respect to age, education, and income level, employment status, sexual orientation, and PrEP use. We sought to minimize differences between pilot study participants and cross-sectional comparison participants by using similar recruitment strategies in terms of advertisements, delivery, and eligibility criteria. Yet the specifics of recruitment differed slightly due to different consent forms, different lengths of participation, and different overall incentive amounts (more study activities were available for pilot participants). There were some differences in participant characteristics, with pilot participants being more likely to come from Chengdu, have recently tested for HIV, and have higher awareness of PrEP. Differences may introduce some bias in comparison, with higher levels of HIV testing and PrEP awareness, perhaps indicating higher pre-existing engagement in prevention care among pilot participants. Exploratory analyses in results assessment may seek to describe the possible impact of these differences. Ultimately, this is a pilot study, and a randomized clinical trial will be needed to determine the efficacy of the intervention if pilot results indicate feasibility and acceptability.

### Limitations

The primary outcome measures of this study rely on self-report data. To account for potential social desirability and recall biases, levels of TFV-DP in the blood will be measured to provide context to the self-report PrEP use data. Given that there was no randomized control group in this pilot study due to funder requirements, there could be selection and secular time biases that are only partially addressed by our inclusion of a 3-month standard-of-care period and a series of repeated cross-sectional surveys among men who have sex with men who did not participate in any HIV prevention study as a comparison.

### Conclusions

This study provides a unique opportunity for potential scalability of a novel intervention by assessing the feasibility and acceptability of integrating an HIV prevention intervention into a widely used GSN app in China. Study results can inform future research to determine the effectiveness of this type of intervention as well as implementation strategies to facilitate future adoption. If successful, app-based interventions have the potential to reach millions of men who have sex with men in China to provide sustainable HIV prevention services.

## Appendix

Multimedia Appendix 1 Focus group discussion guide.

Multimedia Appendix 2 Illustration video of Blued+ app.

Multimedia Appendix 3 Health messages.

Multimedia Appendix 4 Enrollment survey.

Multimedia Appendix 5 TREND checklist.

Multimedia Appendix 6 Peer-review report by ZRG1 AARR-K (03) - Center for Scientific Review Special Emphasis Panel Member Conflict: AIDS and Related Research (National Institutes of Health, USA).

## Abbreviations

AMIS: American Men's Internet Survey
FGD: focus group discussion
GSN: geosocial networking
HBsAg: hepatitis B surface antigen
mHealth: mobile health
PEP: postexposure prophylaxis
PHQ-4: Patient Health Questionnaire for Depression and Anxiety
PrEP: pre-exposure prophylaxis
SHIPP: Sustainable Health Center Implementation PrEP Pilot
TFV-DP: tenofovir diphosphate
UTAUT2: Extended, Unified Theory of Acceptance and Use of Technology

## Data Availability

The datasets are not publicly available as data analysis is ongoing but are available from the corresponding author on reasonable request.
