# Supplementary material for: Integrating a Combination HIV Prevention Intervention Into a Widely Used Geosocial App for Chinese Men Who Have Sex With Men: Protocol for a Single-Arm Pilot and Repeated Cross-Sectional Study
Source: JMIR Res Protoc. 2025 Sep 29;14:e69536. doi: 10.2196/69536 (PMC12519034; doi:10.2196/69536)
Supplement: Multimedia Appendix 1 [file resprot_v14i1e69536_app1.docx]

**Focus Group Discussion Guide**

(Following consent of participants) Welcome, and thank you for being here today. My name is [name], and I am a researcher at [organization]. Today, we’re going to have a discussion about the Blued app and services related to male sexual health. This discussion should last about an hour and a half.

Keep in mind that there are no right or wrong answers. If you feel uncomfortable at any time, you can choose not to answer a question or end the interview. Everything that you say here will be kept confidential. The information may be shared with other member of the research staff, but the information you share with me today will only be used for this research project.

If it is all right with you, I would like to audio record our conversation today. This is so that I can remember everything that you say to me here today without having to write it all down. This recording will be transcribed, so we will be putting all ~~of~~ the words that you say here onto paper. However, there will be no identifying information about yourself or others in the transcriptions. Is it okay with you if I record the discussion?

**Opening/warm-up questions** (5-10min)

1. Can you tell me some experiences that you had were using Blued app? What function do you like/use most frequently?

**Part I** (15-20min)

To start, I would like to walk through the current health-related services in the Blued app. After I show you the feature, we will discuss your thoughts about the portal as it exists today and then how we could modify it to encourage its use.

[Walk-through existing services in the app, especially Blued Welfare and He Health. Share screen, either using the ppt slides or showing them the actual phone screen, to show how each page provides info/services relevant to HIV testing and PrEP] (Screenshots include: Homepage, condom and lubricant, condom, lubricant, PrEP, PrEP-drug, and Testing Kits).

1. Can you tell me your overall thoughts about these services?
   1. Do you know these services are provided on this app before? How do you know them? (Probes: ads on homepage, private message, word of mouth, etc.)
2. Can you tell me about your experiences using these services? [Can change the slide to the specific service that are discussing during the interview.]

***Probe around how frequently participants navigate to the portal, how they are directed to the portal, its ease of use, wording and images.***

- 1. How do you like the wording and images on these pages? (Probes: how easy to understand; How easy to find the service you want; etc.)
  2. How frequently do you use these services? (be specific at each service that they have used)
  3. How do you like these services offering in the app? (Probes: how easy to use these services? How about comparing to these services offering offline?) [Can ask each service separately]
  4. What were your concerns/difficulties when using these services? (Probes: is there anything that worries you or anything that you are unsure about when using these services?) [Can ask each service separately]

1. Other than condoms, lubricant, testing services, and PrEP, are there other services related to HIV that you would like to have on this app? [It’s fine if participants say they don’t know.]

**Part II** (25-30min)

We are developing an intervention to increase the uptake of HIV pre-exposure prophylaxis. Pre-exposure prophylaxis (PrEP) is taking an antiretroviral pill, also called Truvada, every day for months or years to reduce a person’s chance of getting HIV. Research has shown PrEP is up to 99% effective in preventing HIV when taken daily. To get PrEP, a person needs to go to the doctor, get tested for HIV every three months, and pay for the medication, which could cost approximately CNY 2000 for the imported drug (Truvada) or CNY 500 for the domestic drug (Taihe) each month. PrEP is not currently widely available in China.

1. What types of interventions, functions, or services do you think would be most helpful to encourage people to start PrEP?
   1. What information would you like to know about PrEP? (Probes: how about efficacy, cost, side effect, lab tests and clinical follow-ups, users experience stories, etc.)
   2. In what way you would like to be directed to the relevant page(s)? (Probes: homepage ads, opening ads, private message, livestreams, etc.)
   3. Can you describe ways this portal could be improved to make you more likely to use that type of intervention?
2. I am now going to review some functions for PrEP promotion we have been thinking about.
   1. **PrEP education information**
      1. Can you tell me your thoughts about this component? / Can you tell me your initial reactions to this component being included in the app?
      2. If this feature were to be added to the app, can you describe what the feature should look like? [let participants answer freely before asking the following specific questions]
         1. What PrEP education information that you would like to see? (Probes: what is PrEP; PrEP regimens; Is PrEP right for you; how to use PrEP; PrEP efficacy and safety; clinical follow-up and monitoring; potential side effect, cost, etc.)
         2. How do you like this information to present? (Probes: formal and professional, educational, easy to understand, etc.)
         3. How should this feature be incorporated? Where would it best be placed in the app.
      3. What concerns do you have for this feature/component?
   2. **PrEP screen quizzes to see if PrEP is right for you**
      1. Can you tell me your thoughts about this component? / Can you tell me your initial reactions to this component being included in the app?
      2. If this feature were to be added to the app, can you describe what the feature should look like? [let participants answer freely before asking the following specific questions]
         1. What suggestions that you would like to see after the screening? (Probes: simple categories of eligible or not eligible, or with explanations, or link to counseling services, etc.)
         2. Would you want the suggestions to be sent through private messaging, or email, so that you can find them in your inbox later?)
         3. How should this feature be incorporated? Where would it best be placed in the app.
      3. What concerns do you have for this feature/component?
   3. **Referrals to no-cost PrEP services: a clinician visit, lab tests, and pharmacy services** (explain when needed: connecting you with appointed physicians in the tier three hospital who can offer counseling services of PrEP and prescribe PrEP.)
      1. Can you tell me your thoughts about this component? / Can you tell me your initial reactions to this component being included in the app?
      2. If this feature were to be added to the app, can you describe what the feature should look like? [let participants answer freely before asking the following specific questions]
         1. How would you like to connect with the physician? (Probes: text messaging with the physician within this app, phone calls, offline appointment; anonymous; etc.)
         2. What type of physician would you expect? (Probes: working in tier three hospital, STI specialist, MSM friendly, etc.)
         3. How should this feature be incorporated? Where would it best be placed in the app.
      3. What concerns do you have for this feature/component?
   4. **Reminder functions** (explain when needed: there will be notifications reminding you to take the medicine and regular clinician visits.
      1. Can you tell me your thoughts about this component? / Can you tell me your initial reactions to this component being included in the app?
      2. If this feature were to be added to the app, can you describe what the feature should look like? [let participants answer freely before asking the following specific questions]
         1. How would you like this message to be sent through? (Probes: text message, private message through the app, phone call, etc.)
         2. How should this feature be incorporated? Where would it best be placed in the app.
      3. What concerns do you have for this feature/component?
   5. **PrEP-experienced peer service: ask questions of and talk to your peers who are experienced PrEP users**  (explain when needed: peer refers to people who is using PrEP)
      1. Can you tell me your thoughts about this component? / Can you tell me your initial reactions to this component being included in the app?
      2. If this feature were to be added to the app, can you describe what the feature should look like? [let participants answer freely before asking the following specific questions]
         1. How would you like this message to be sent through? (Probes: private and anonymous messages, create a PrEP-user community, etc.)
         2. How likely that you would be willing to use this feature/component?
         3. How should this feature be incorporated? Where would it best be placed in the app.
      3. What concerns do you have for this feature/component?
   6. **Interest in using alternative PrEP regimens: long-acting injectable (LAJ) PrEP** (which could be approved in China soon)
      1. (Explain as needed: The proposing long-acting injectable form of PrEP is structured as a lead-in phase with an oral version of the injectable medication to ensure no serious adverse events, then commencement of the injectable medication every 8 weeks. Once the medication is stopped due to patient or provider preference, it may be recommended to continue a “tail” with oral Truvada for the following year to prevent the development of resistance.)
      2. Can you tell me your initial reactions to this component being included in the app?
      3. How likely that you would want to use this form of PrEP if you are eligible?
      4. What information about LAJ PrEP would you want to know?
      5. What concerns do you have for LAJ PrEP?
   7. **Intermittent** **PrEP** **interest**
      1. Explain as needed: The proposing intermittent PrEP is structured as 2-1-1 dosing of oral medication. Once the medication is stopped due to patient or provider preference, it may be recommended to continue a “tail” with oral Truvada for the following year to prevent the development of resistance.
      2. Can you tell me your initial reactions to this component being included in the app?
      3. How likely that you would want to use this form of PrEP if you are eligible?
      4. What information about intermittent PrEP would you want to know?
      5. What concerns do you have for intermittent PrEP?
3. Our plan so far includes several components: PrEP education information, PrEP screen quizzes to see if PrEP is right for you, referrals to a doctor for PrEP, no-cost PrEP where drug, lab tests, and clinician visit are covered, reminder functions, and peer-to-peer messaging.
   1. Do you think these services should be organized in a central system (e.g. PrEP headquarters) or spread throughout the app, or other? How should we organize these components in a central/separated way (depends on participants answer), can you tell me more details or more specifically? (e.g. in what order these components should show up, or which components should be bundled together) Why do you think this way is better?
   2. Tell me about your thoughts in general?
   3. Are there any additional features we should add?
   4. Which components you would be most likely to use? Which components you would be least likely to use?

This is all we would like to cover today. You have each given us some important information today! I appreciate your participation. What questions do you have for me before we finish?
