# Supplementary material for: Integrating a Combination HIV Prevention Intervention Into a Widely Used Geosocial App for Chinese Men Who Have Sex With Men: Protocol for a Single-Arm Pilot and Repeated Cross-Sectional Study
Source: JMIR Res Protoc. 2025 Sep 29;14:e69536. doi: 10.2196/69536 (PMC12519034; doi:10.2196/69536)
Supplement: Multimedia Appendix 3 [file resprot_v14i1e69536_app3.docx]

**Health Messages**

| **Category** | **Message** | **Page directed by the hyperlink** |
| --- | --- | --- |
| Condom Use | Condoms significantly reduce the risk of getting HIV and other STDs when used correctly and consistently. | Condom Ordering Page |
| Condom Use | When using compatible lubricant, condoms failed less than 1% of the time for anal sex. Find quality lubricants and condoms today! | Lubricant Ordering Page |
| Condom Use | Want to reduce your risk of having a condom break or slip off? Water- or silicone-based lubricants are safe to use with condoms and make sex more enjoyable | Lubricant Ordering Page |
| Condom Use | Got a hot date? Don't go unprepared. Get some free condoms and lube today! | Condom Ordering Page |
| Condom Use | For many men, an active and healthy sex life includes consistent condom use. Latex condoms remain one of the most successful ways to prevent HIV and other STDs. | Condom Ordering Page |
| Condom Use | Going out? Make sure you have everything you need: the phone, keys, wallet, gum, condoms and lube. | Condom Ordering Page |
| Engagement in Healthcare | Talking about sex with a health care provider may be uncomfortable, but you can always find a safe place here at Blued+ chatting with our health care provider. | Chat with Doctor |
| Engagement in Healthcare | Invest in your health by knowing your HIV status. Click here to see the different ways to get tested. | HIV Testing Ordering Page |
| HIV Testing | Not only can this app show you the nearest place to get an HIV test, but it can also help you choose the test that's right for you. Check here. | HIV Testing Ordering Page |
| HIV Testing | Haven't been tested in the last 6 months? Find the test that fits in your schedule and get tested today. | HIV Testing Ordering Page |
| HIV Testing | People living with HIV who are on regular treatment live a healthy life. Getting an HIV test is the only way to know your status. | HIV Testing Ordering Page |
| HIV Testing | It is recommended by Chinese CDC that sexually active men get tested for HIV every 6 months, and as often as every 3 months if preferred. Find out more information here. | HIV Testing Ordering Page |
| HIV Testing | Not sure which HIV test is right for you? Rapid tests can give you results in minutes, and there are more ways than ever to know your status. Find out what test is right for you. | HIV Testing Ordering Page |
| HIV Testing | Many men talk about HIV with their partners and friends. Making HIV testing part of your routine, and encouraging people around you to also get tested! | HIV Testing Ordering Page |
| PrEP Use | Wondering how you can prevent HIV? PrEP might be right for you! Talk to a Blued+ doctor today. | Chat with Doctor |
| PrEP Use | Could PrEP be right for you? Chat with our Blued+ provider. (link) | Chat with Doctor |
| PrEP Use | Worried about the side effects of PrEP? Most people don't experience PrEP side effects after 1 month and side effects usually go away over time. | Homepage |
| PrEP Use | As a valued user of Blued+, you can get PrEP at no cost for the doctor's visit or the medication. Take advantage of this today! | Chat with Doctor |
| PrEP Use | Don't let the cost stop you from getting PrEP! We can offer free PrEP appointments for you. Check out with your local health care provider! | PrEP Application Page |
| PrEP Use | Using PrEP consistently provides nearly 100% protection against HIV. Make the next step of your PrEP journey today! | PrEP Application Page |
| PrEP Use | Heard about PrEP? Pre-exposure prophylaxis or "PrEP" is a pill taken daily by HIV-negative men to prevent HIV infection. | Homepage |
| PrEP Use | PrEP and condoms offer a lot of protection. Using them together increases success in preventing HIV and STD infections. | Homepage |
| PrEP Use | if you ordered PrEP in Blued+ but haven’t had your physical exam, do it today, to protect yourself tomorrow! Blued+ will reimburse your cost for the physical exam. | PrEP Application Page |
| PrEP Use | You can significantly reduce the risk of getting HIV by sticking to your daily PrEP routine. Setting a reminder can help! | PrEP Application Page |
| PrEP Use | PrEP use is becoming more and more common in our community. Find a health care provider near you! | PrEP Application Page |
| Self-Support | To love oneself is the beginning of a lifelong romance. —Oscar Wilde | Homepage |
| Self-Support | Loving yourself is literally an attractive quality. True self love is like a magnet that draws other people into your world and increases the value of connections. | Homepage |
| Self-Support | Don't judge your insides by other people's outsides. Lots of people whose lives look perfect are struggling behind closed doors. Focus on being the best you. | Homepage |
| Self-Support | Pain and hurt feelings are inevitable parts of connecting. But you can avoid suffering by knowing someone's rejection doesn't "mean" anything about you. | Homepage |
