# Supplementary material for: Integrating a Combination HIV Prevention Intervention Into a Widely Used Geosocial App for Chinese Men Who Have Sex With Men: Protocol for a Single-Arm Pilot and Repeated Cross-Sectional Study
Source: JMIR Res Protoc. 2025 Sep 29;14:e69536. doi: 10.2196/69536 (PMC12519034; doi:10.2196/69536)
Supplement: Multimedia Appendix 4 [file resprot_v14i1e69536_app4.docx]

**Enrollment Survey**

**Contents**

[**Introduction** 7](#_Toc182996963)

[**What is the purpose of this study?** 7](#_Toc182996964)

[**What will I be asked to do?** 7](#_Toc182996965)

[**Risks and Discomforts** 9](#_Toc182996966)

[**New Information** 10](#_Toc182996967)

[**Benefits** 10](#_Toc182996968)

[**Withdrawal from the Study** 11](#_Toc182996969)

[**Consent** 11](#_Toc182996970)

[**Sociodemographic Questions** 14](#_Toc182996971)

[**Race and Ethnicity** 14](#_Toc182996972)

[**Education** 14](#_Toc182996973)

[**Employment** 14](#_Toc182996974)

[**Income** 14](#_Toc182996975)

[**Sexual Identity** 14](#_Toc182996976)

[**A.** **Relationship Status** 16](#_Toc182996977)

[**B.** **Sexual History** 16](#_Toc182996978)

[**C.** **HIV/STI Testing History** 20](#_Toc182996979)

[**D.** **PrEP Knowledge/Attitudes** 21](#_Toc182996980)

[**E.** **PrEP Intention and use** 21](#_Toc182996981)

[**F.** **PrEP formulation preferences** 23](#_Toc182996982)

[**G.** **HIV Knowledge** 25](#_Toc182996983)

[**H.** **PrEP Stigma** 26](#_Toc182996984)

[**I.** **Mental Health** 28](#_Toc182996985)

[**J.** **Order History** 28](#_Toc182996986)

**Emory University**

**Consent Script and Information Sheet**

**For Research Study Screening**

**Study Title:** Integrating a Combination HIV Prevention Intervention into a Widely-Used Geosocial App for Chinese MSM

**Principal Investigator:** Aaron Siegler, PhD, MHS; Department of Behavioral Sciences and Health Education, Emory University

**Funding Source:** National Institutes of Health

**Introduction and Study Overview**

Thank you for your interest in this study. To see whether you may be a candidate for this study, we need to ask you for some information about yourself. But first, let me tell you about this screening consent and what we will do with your information.

1. This screening interview will take about 5 minutes.

2. You can also stop the screening questionnaire at any time. This is completely voluntary.

3. We can send you an information sheet about this screening, along with the screening questions, if you would like. We will also give you a form you can send in later if you change your mind and want us to remove your information from our records.

4. We will ask you about your demographics and sexual behaviors and will record this information in a database containing information from others who have also shown interest in the study.

5. This information will only be used for the research study you are interested in.

6. The only risk to you in this screening is a potential loss of privacy. However, your privacy is very important to us and we will be very careful with your information.

7. The following persons or groups may use and/or disclose your information for this study:

· The Principal Investigator and the research staff

· The National Institutes of Health, who funds this research, and people or companies they use to carry out the study

· Any government agencies who regulate the research including the Office of Human Subjects Research Protections, the Emory Institutional Review Board, the Institutional Review Board of the National Center for AIDS/STD Control and Prevention (NCAIDS), and the Emory Office of Research Compliance.

**Contact Information**

If, at any time, you have questions about this screening process, your rights as a research participant, or if you have questions, concerns or complaints about the research you may contact the study Principal Investigator, Aaron Siegler, Dr. Mi Guodong at Blued, the Emory Institutional Review Board, or the Institutional Review Board of NCAIDS.

Dr. Aaron Siegler, [asiegle@emory.edu](mailto:asiegle@emory.edu)

Dr. Mi Guodong, [migd@outlook.com](mailto:migd@outlook.com)

Emory Institutional Review Board at +1 404-712-0720 or by email at [irb@emory.edu](mailto:irb@emory.edu)

Institutional Review Board of NCAIDS at [mengchi@chinaaids.cn](mailto:mengchi@chinaaids.cn)

You can also stop the screening interview at any time. This is completely voluntary.

**Consent**

__ I consent to being screened for this study online

__ I do NOT consent to being screened for this study online

***[If Participant Consents for Screening, participants is directed to online screener.***

***[If Participant does Not Consent for Screening,*** participant is directed to a webpage reading:

“You have indicated that you do not consent to taking the eligibility screener for our study. We require all respondents to consent to take the eligibility survey in order to participate in the study, so we cannot allow you to participate. Thank you for your interest in our study.

---Page break---

**Eligibility Screener**

Hello and thank you for your participation in this Blue+ survey!

We are going to be asking questions about various topics including your background and life experience, healthcare, HIV prevention, your sexual partners and relationships, alcohol and substance use.

Helpful tips:

- Questions marked with a red asterisk (*) are required questions that you must answer to move forward.
- This is a forward-only survey. When you finish a page, proceed to the next page by clicking the “Next” button. You may not go backwards to pages you already complete. Please don’t use the back button on your browser.

Your privacy is important to us. All information you provide in this survey will be held confidentially. Your answers will not be linked with your name. If you consent, please click the “next” button below to continue to the survey.

---Page break---

1. What is your current age? *

[_____] years old Eligible if ≥ 18 years

1. In which city do you live **mainly** in the past 2 years? *
   1. Beijing
   2. Chengdu
   3. Guangzhou
   4. Shanghai
   5. Shenzhen
   6. Wuhan
   7. Other
2. What was your sex assigned at birth? *
   1. Male
   2. Female
   3. Other (please describe):
3. Have you ever had any positive HIV test? * (NHBS)
4. Yes
5. No
6. Are you currently enrolled in any other HIV prevention studies? *
7. Yes
8. No
9. In the past 6 months, have you had anal sex with a male partner? *
   1. Yes
   2. No
10. In the past 6 months, have you ever engaged in anal sex with a male partner without using condoms? * (Consensus statement) (if Yes, then eligible regardless the response to question h, i, j, k, l, m)
11. Yes
12. No
13. In the past 6 months, have you ever engaged in vaginal or anal sex with a female partner without using condoms? * (Consensus statement) (if Yes, then eligible regardless the response to question g, i, j, k, l, m)
14. Yes
15. No
16. In the past 6 months, have you ever injected drugs that were not prescribed by a clinician or shared needles with others? * (Consensus statement) (if Yes, then eligible regardless of the response to question g, h, j, k, l, m)
17. Yes
18. No
19. In the past 6 months, have you ever had a sexual partner who was infected with HIV? * (Consensus statement) (if Yes, then eligible regardless of the response to question g, h, i, k, l, m)
20. Yes
21. No
22. In the past 6 months, are you newly diagnosed with sexual transmitted diseases (e.g., syphilis, gonorrhea, chlamydia)? * (Consensus statement) (if Yes, then eligible regardless of the response to question g, h, i, j, l, m)
23. Yes
24. No
25. Have you previously used PrEP/PEP in the past 6 months? * (Consensus statement) (if Yes, then eligible regardless of the response to question g, h, i, j, k, m)

Pre-exposure prophylaxis (PrEP) is taking an antiretroviral pill, also called Truvada, every day for months or years to reduce a person’s chance of getting HIV. Post-exposure prophylaxis (PEP) means taking medicine to prevent HIV after a possible exposure. PEP should be used only in emergency situations and must be started within 72 hours after a recent possible exposure to HIV.

1. Yes
2. No
3. Are you interested in initiating PrEP? (if Yes, then eligible regardless of the response to question g, h, i, j, k, l)
   1. Yes
   2. No

Thank you for your participation. You have completed the screening.

[Click complete to see if you are eligible]

---Page break---

**Emory University**

**Consent to be a Research Subject**

**Title**: Integrating a Combination HIV Prevention Intervention into a Widely-Used Geosocial App for Chinese MSM

**Principal Investigator:** Aaron Siegler, PhD, MHS; Department of Behavioral Sciences and Health Education, Emory University

**Funding Source:** National Institutes of Health

## **Introduction**

You are being asked to be in a public health research study. This study is being done by Dr. Mi Guodong at Blued and Dr. Aaron Siegler from Emory University’s Rollins School of Public Health. This form is designed to tell you everything you need to think about before you decide if you want to be a part of the study. **It is entirely your choice. If you decide to take part, you can change your mind later on and withdraw from the research study.** **You can skip any questions that you do not wish to answer.** The decision to join or not join the research study will not cause you to lose any medical benefits.

Before making your decision:

- Please carefully read this form or have it read to you
- Please ask questions about anything that is not clear

You can save a copy of this consent form to keep. Feel free to take your time thinking about whether you would like to participate. You may wish to discuss your decision with family or friends. Do not sign this consent form unless you have had a chance to ask questions and get answers that make sense to you. By signing this form, you will not give up any legal rights.

## **What is the purpose of this study?**

The purpose of this study is to test a smartphone app designed to promote healthy behaviors in men who have sex with men. This will be done through information, health messaging, periodic screenings, and prevention resources. We will ask participants to tell us about their thoughts on the app and its use. We will also ask participants about their sexual health and history in order to determine whether using the app has an effect on sexual health outcomes.

## **What will I be asked to do?**

If you choose to be in this study, we will ask you to participate in a series of activities over the course of 15 months.

These activities will include filling out surveys about your sexual health and behavior, and interacting with a smartphone app Blued+. The study activities are described individually below:

Six 45-minute surveys

If you choose to be in this study, we will ask you to complete a baseline survey through the Blued app on your own electronic device (i.e. smartphone) to establish some basic facts about you for our study. These facts include your sexual history, medical history, behaviors, and attitudes. We will also ask you to complete a series of follow-up surveys for our study. These will be delivered to you through the Blued app every three months (i.e. 3, 6, 9, and 12 months) after today. These follow-up surveys will repeat many of the questions from the baseline survey to determine whether your behaviors, beliefs, and attitudes have changed.

App Interactions

Three months from today, you will also be asked to interact with a version of the Blued app with additional health resources. You will receive a version of the Blued app with health education, health messaging, and health quizzes delivered through the app. You will be able to order health HIV prevention materials such as condoms, lubricants, and HIV tests through the App. All HIV prevention materials will be provided at no cost to you. You will be expected to interact with the app regularly. As you use the app, it will collect data about that use.

Pre-Exposure Prophylaxis Sub-study

You will be asked to participate in a sub-study of Blued. This sub-study offers pre-exposure prophylaxis (PrEP), also known as Truvada or FTC/TDF. PrEP is a type of antiretroviral tablet that is taken to lower the rate of new HIV infections when used with other HIV prevention tools. Truvada has been shown to be >99% effective in preventing HIV in MSM who take the medication as directed. Used this way, PrEP is not meant to treat any illnesses that you may have, but rather to help keep you from getting HIV. You are not required to take PrEP if you join this larger Blued study. However, if you are interested, we can help you to schedule an online or in-person interview with the study doctor for PrEP initiation, and if you meet the requirements, we will help you schedule a visit with a study doctor or nurse who prescribes PrEP. They will use your blood sample to check if you are eligible. This will make sure your kidneys function by testing your creatinine levels in your blood. You will also be tested for HIV. You will have additional regular study visits if you choose to start PrEP. All participants in this study will have the option of receiving PrEP free of charge. There will be no cost to you for these services.

The purpose of this sub-study is to study the attitudes and behaviors of local men who are eligible to start taking PrEP, as offered as part of an HIV prevention package through the Blued app. If you join this sub-study today, you will be told about PrEP. If you are eligible, you will be scheduling online or in-person PrEP appointment through Blued App and the study doctor will give you a prescription for Truvada (FTC/TDF). We will then provide you with a prescription and mail PrEP drug to your address .

To ensure your safety, you will be asked to come back to the test site every 3 months after starting PrEP for HIV testing, STI testing, and other testing. This is the standard for men who take PrEP. This means that you may expect to come to the site for about 4 extra visits over the course of the year. During your visits, you will meet with the study staff and/or study doctor who will talk to you about how you have been feeling since starting the medication. The study doctor will review your medical history and we will collect some blood from you. Each blood draw will be about 15mls (1 tablespoon full). The blood sample will be used to assess your HIV status and measure creatinine levels to ensure your kidneys are healthy. An additional blood sample may also be collected to determine how much of the PREP drug, Truvada, is present in your blood. During your regular study surveys, you will be asked questions about your experiences taking PrEP. This may also involve a more detailed in person interview with one of the study doctors.

In order for the doctor to be able to renew your prescription for PrEP, it is important to come to your visits every 3 months. The information about your health learned at these visits will be used by the doctor to renew your prescription. At the end of the 12-month period of Blued, the study will stop providing prescriptions for PrEP and will stop PrEP-related testing.

There may be minor discomfort from blood draws. The blood draws may cause bruising. There is a slight risk of an infection where the blood was drawn.

There may be side effects from the study drug. There is a chance that you could have nausea, vomiting, and diarrhea when taking the drug. You may also have some weight loss. The use of the drug may less commonly cause severe side effects. More serious effects may include allergic reactions (rash; itching; difficulty breathing; swelling of your face, lips, throat, or tongue). They may also include bone pain; mood changes; muscle pain or weakness; severe dizziness; symptoms of kidney problems (e.g., increased or decreased urination, increased thirst) and symptoms of liver problems (e.g., yellowing of the skin or eyes; dark urine; pale stools; persistent loss of appetite). Please call the study staff if you are having any concerns. If needed, we will have the study doctor call you back. If you are having a medical emergency, please seek immediate medical attention.

If you become HIV infected while taking Truvada, there is a chance that the HIV virus could develop resistance to Truvada. The best way to avoid this is to take your medication strictly following the prescription.

Please keep the study drug out of the reach of children or anyone else who may not be able to read or understand the label. Do not let anyone else take the study drug besides you.

##### There will be no additional compensation for participating in this sub-study.

There will be no costs to you for participating in this study, other than basic expenses like

transportation.

This section of consent is designed to tell you everything you need to think about before you decide if you want to take PrEP or not. **It is entirely your choice. If you begin taking PrEP now, you can change your mind later on and stop taking it.** Either way, you will remain enrolled in the larger Blued Study.

## **Risks and Discomforts**

There are minor risks associated with this study. Some of the questions in the survey are personal, and may make you uncomfortable. We hope you will answer all questions to the best of your ability. You can choose not to answer any question that makes you uncomfortable. We will keep information about your HIV and STI testing, and your responses to the survey questions. Although we will take steps to reduce the chance, there is a small chance that someone other than study staff might see your study information. More information about how we will protect your confidentiality is below.

There is a possibility that someone may see the mobile app on your device. Because this app provides information about STIs, HIV, and sexuality, there is a risk of breach of privacy. To prevent this, we recommend closing out of the app and/or locking your mobile phone when you are not interacting with the app.

## **New Information**

It is possible that the researchers will learn something new during the study about the risks of being in it. If this happens, they will tell you about it. Then you can decide if you want to continue to be in this study or not. You may be asked to sign a new consent form that includes the new information if you decide to stay in the study.

## **Benefits**

This study is not designed to benefit you directly. However, you may benefit from participating because the mobile app will provide information and HIV prevention resources.

This study may also indirectly benefit you because we may learn about how to promote prevention services that can help reduce the health burden of HIV and STIs among men who have sex with men.

##### **Compensation**

You will get ¥100 ($15) for each of the six study survey you complete (at 0, 3, 6, 9, 12, and 15 months). If you do not finish the study, you will be paid for the surveys you have completed. If you complete all six study surveys, you will receive an extra ¥400 ($60), for a total of ¥ 1000 ($160).

##### **What are my other options?**

If you decide not to enter this study, there is care available to you outside of this research. You should discuss this with the researchers if you have concerns or want to know about other options.

###### **How will you protect my private information that you collect in this study?**

All information about you obtained from this research study will be kept as confidential as possible. Your personal information may be disclosed if required by law. Any publication of this study’s results will not use your name or identify you personally in any way. The study staff may use your personal information to verify that you are not in any other research studies.

Certain offices and people other than the researchers may look at study records. Government agencies and Emory University employees overseeing proper study conduct may look at your study records. These offices include the Office for Human Research Protections, the Emory Institutional Review Board, the Institutional Review Board of the National Center for HIV/STD Control and Prevention (NCAIDS), and the Emory Office of Compliance. Study funders may also look at your study records. Emory will keep any research records we create private to the extent we are required to do so by law. A study number rather than your name will be used on study records wherever possible. Your name and other facts that might point to you will not appear when we present this study or publish its results.

## **Withdrawal from the Study**

You have the right to leave this study at any time without penalty. If you leave the study before the final planned study visit, the researchers may ask you to have some of the final steps done. The researchers also have the right to stop your participation in this study without your consent for any reason, especially if they believe it is in your best interest or if you were to object to any future changes that may be made in the study plan.

**Contact Information**

Contact the China study coordinator: Yu Fei at +86 18980952400 or [yufei@blued.com](mailto:yufei@blued.com)

- if you have any questions about this study or your part in it,
- if you have questions, concerns or complaints about the research

Contact the Emory Institutional Review Board at +1 404-712-0720 or [irb@emory.edu](mailto:irb@emory.edu) or the IRB of NCAIDS, Ms. Liu Mengchi at [mengchi@chinaaids.cn](mailto:mengchi@chinaaids.cn):

- if you have questions about your rights as a research participant.
- if you have questions, concerns or complaints about the research.
- You may also let the Emory University IRB know about your experience as a research participant through our Research Participant Survey at <http://www.surveymonkey.com/s/6ZDMW75>.

## **Consent**

Being in this study is entirely your choice. You have the right to refuse to participate or to stop at any time. Please print a copy of this form for your records.

If you agree to the above information and would like to be in the study, please sign your name using mouse or touch pad, and then type in your name below. *

I understand that checking this box constitutes a legal signature confirming that I have read the consent form, and agree to participate in the Blued study. *

- Legally sign document
- Do NOT legally sign document

---Page break---

**Blued+ Pilot Study Consent Quiz**

Hello! You have just finished reading the Blued+ consent, and It's now time to test your knowledge! Do not worry, the quiz only has 5 questions, and it just helps to understand how well you know the consent, and make sure you recognize all your rights so we could help you better!

**Q1. How long is the study?**

1. 3 month, surveys every month
2. 12 months, surveys every 3 months
3. 15 months, surveys every 3 months
4. 18 months, survey every 3 months

A1. 15 months, surveys every 3 months

Explanation: The study will last 15 months and surveys will be administered every 3 months. The study will end in October 2023.

**Q2. When will the Blued+ services be available to me?**

As soon as I complete study initiation today

1 month after study initiation

3 months after study initiation

6 months after study initiation

A2. 3 months after study initiation

Explanation: Blued+ App service will be available 3 months from today (October 2022). 2022). 3 months between today and the intervention is called the Stand of Care period, which serves as an baseline of the study and allows you to access the existing Blued app and local health services as usual. You will be asked to take the second survey and your blue app will be automatically updated to the enhanced version including Blued+ service in September 2022.

**Q3. What functions does the Blued+ service have?**

Ordering free condoms and lubricants

Ordering free at home HIV tests

Getting free PrEP after a free PrEP initiation doctors visit at a local clinic

All of above

A3. All of above

Explanation: Using the Blued+ service, you will be able to order free condoms, lubricants, and at home HIV testing kits. Also, if you are interested, you are highly welcome to initiate PrEP.

**Q4. How much do I need to pay to receive the HIV prevention intervention goods provided by the study?**

1. ¥640 ($100)
2. ¥320 ($50)
3. ¥125 ($20)
4. ¥0 ($0)

A2: ¥0 ($0)

Explanation: researchers want to understand the impact of increasing access to these prevention services by removing cost as a barrier, so all study components will be provided to you at no cost.

**Q5. How much will I get paid by the study if I stay till the end and complete all the surveys?**

1. ¥1000 ($160)
2. ¥750 ($120)
3. ¥320 ($50)
4. ¥125 ($20)

A4: ¥1000 ($160)

Explanation: In order to thank you for donating your time taking the surveys, we will provide ¥100 ($15) for each survey you take, and an extra of ¥400 ($60) if you complete all 6 surveys at the end—that sums up to ¥1000 ($160)!

**Q6: Can I quit when I do not want to participate anymore?**

1. Yes, you can quit at any point of the study
2. No, you have to stay till the end of the study

A5: Yes, you can quit at any point of the study

Explanation: Once you have decided to join the study, you can quit any time when you don't feel like participating anymore. It’s totally your choice! Just send us a message and let us know!

**Thank you for taking the consent quiz! If you have more questions, please contact us!**

---Page break---

# **Sociodemographic Questions**

## **Race and Ethnicity**

1. Which ethnic group do you consider yourself to be in? Select all that apply. *
2. Han
3. Zhuang
4. Hui
5. Man
6. Uyghurs
7. Miao
8. Yi
9. Tujia
10. Tibetan
11. Mongols
12. Other (please describe):

## **Education**

1. What is the highest level of education (school) that you completed? *
2. Post graduate and above
3. College or university
4. Associate’s degree and/or technical school
5. High school
6. Middle school
7. Less than middle school

## **Employment**

1. What best describes your employment status? *
2. Employed full-time
3. Employed part-time
4. Full-time student
5. Part-time student
6. Unemployed
7. Other (please describe):

## **Income**

1. What is your monthly income (RMB)? *
2. <3000
3. 3000-6999
4. 7000-10000
5. >10000

## **Sexual Identity**

1. What is your current sexual identity? *
2. Gay, homosexual, same gender loving
3. Straight or heterosexual
4. Bisexual
5. Other, specify

**Covid Quarantine Impact**

1. In the past 3 months, have you had a quarantine at your region or neighborhood that you were unable to leave home for a period of time?
   1. Yes
   2. No

---Page break---

1. **Relationship Status**
2. Do you currently have a main sexual partner or boyfriend? *
3. Yes
4. No (Logic: Jump to B1)
5. How do you and your main sexual partner or boyfriend handle sex outside of your relationship? *
6. We only have sex with each other
7. We are in an open-relationship
8. We haven’t discussed/don’t know
9. What is the HIV status of your main sexual partner or boyfriend? *
10. HIV-positive
11. HIV-negative
12. Don’t know

---Page break---

1. **Sexual History**
2. In the **past 6 months**, with how many male partners have you had anal sex? * (Element)

1. In the **past 3 months**, with how many male partners have you had anal sex?

1. In the **past 3 month**s, what are the HIV statuses of the [B2] male partners you had anal sex with?
   1. HIV+
   2. HIV-
   3. HIV status unknown
2. As far as you know, how many of the [B2] male partners have you had anal sex with who had a **positive HIV status**? * (Element) [if B3=a]

1. As far as you know, how many of the [B2] male partners have you had anal sex with who had a **negative** **HIV status**? * [if B3=b]

1. As far as you know, how many male of the [B2] partners have you had anal sex with who had an **unknown** **HIV status**? * [if B3=c]

1. In the past 3 months, of the [B4] partners that you said are HIV positive, how many do you know to be on anti-retroviral treatment (ART)?: * [If B3=a]

1. In the past 3 months, of the[B5] partners that you said are HIV negative, how many partners do you know to be on PrEP?: *[If B3=b]

---Page break---

1. In the past 3 months, did you have receptive anal sex (you were the bottom)? *
2. Yes
3. No (Logic: Jump to B14)
4. In the past 3 months, how often did your partner use condoms?
5. Always (100%) (Logic: Jump to B14)
6. Often (50-99%)
7. Sometimes (1-50%)
8. Never (0%) (Logic: Jump to B14)
9. In the past 3 months, how many sex acts did you have as receptive with a male partner? *

1. Of those [answer from B11] receptive sex acts, how many times did your partner use condoms? *

1. The last time you had receptive anal sex, did your partner wear a condom? *
2. Yes
3. No

---Page break---

1. In the past 3 months, did you have insertive anal sex (you were the top) with a male partner?
2. Yes
3. No (Logic: Jump to B19)
4. In the past 3 months, how often did you use condoms?
5. Always (100%) (Logic: Jump to B19)
6. Often (50-99%)
7. Sometimes (1-50%)
8. Never (0%) (Logic: Jump to B19)
9. In the past 3 months, how many sex acts did you have as insertive with a male partner? *
10. Of those [answer from B16] insertive sex acts, how many times did you use condom? *
11. The last time you had insertive anal sex with a male partner, did you wear a condom? *
12. Yes
13. No

---Page break---

1. In the past 3 months, did you have vaginal sex with a female partner?
2. Yes
3. No (Logic: Jump to B24)
4. In the past 3 months, how often did you use condoms when you have vaginal sex with a female partner?
5. Always (100%) (Logic: Jump to B24)
6. Often (50-99%)
7. Sometimes (1-50%)
8. Never (0%) (Logic: Jump to B24)
9. In the past 3 months, how many vaginal sex acts did you have with a female partner? *

1. Of those [answer from B21] vaginal sex acts, how many times did you use condom? *

1. The last time you had vaginal sex with a female partner, did you wear a condom? *
2. Yes
3. No

---Page break---

1. In the past 3 months, did you have anal sex with a female partner? *
2. Yes
3. No (Logic: Jump to B29)
4. In the past 3 months, how often did you use condoms when you have anal sex with a female partner?
5. Always (100%) (Logic: Jump to B29）
6. Often (50-99%)
7. Sometimes (1-50%)
8. Never (0%) (Logic: Jump to B29)
9. In the past 3 months, how many anal sex acts did you have with a female partner? *

1. Of those [answer from B26] anal sex acts with a female partner, how many times did you use condom? *

1. The last time you had anal sex with a female partner, did you wear a condom? *
2. Yes
3. No

---Page break---

1. In the past 3 months, have you received any free condoms (for example, from CBOs, VCT, community activities, etc.)? *
2. Yes
3. No (Logic: Jump to B31)
4. Have you used these free condoms? *
5. Yes
6. No
7. In the past 3 months, have you received any free lubricant (for example, from CBOs, VCT, …, etc.)? *
8. Yes
9. No (Logic: Jump to C1)
10. Have you used the free lubricant? *
11. Yes
12. No

---Page break---

1. **HIV/STI Testing History**

**This next section will ask some questions about your experiences with HIV and STI testing. Common sexually transmitted infection (STI) include syphilis, gonorrhea, chlamydia, herpes, and genital warts.**

**Everything you tell us today is confidential and will be used for study purposes only. We appreciate your honest answers to better understand HIV and STI testing patterns.**

1. Have you ever had an HIV test? * (NHBS)
2. Yes
3. No (Logic: Jump to C7)
4. When was the last time you were tested for HIV? *
5. 3 or fewer months ago
6. 4 to 6 months ago (Logic: Jump to C5)
7. 7 to 12 months ago (Logic: Jump to C5)
8. More than 12 months ago (Logic: Jump to C5)
9. In the **past 3 months,** how many times did you test for HIV? *
10. 1 time
11. 2 times
12. 3 times
13. More than 3 times
14. In the **past 3 months,** what type of HIV testing did you get? Check all that apply. * (Logic: Jump to C6)
15. HIV screening test at hospital or local CDC
16. HIV confirmatory test at hospital or local CDC
17. HIV test at CBO (e.g., Blued, community organizations)
18. At-home HIV self-test accessed through Blued
19. At-home HIV self-test accessed outside of Blued
20. The last time you tested for HIV, what type of HIV testing did you get? *
21. HIV screening test at hospital or local CDC
22. HIV confirmatory test at hospital or local CDC
23. HIV test at CBO (e.g., Blued, community organizations)
24. At-home HIV self-test accessed through Blued
25. At-home HIV self-test accessed outside of Blued
26. The last time you tested for HIV, what was your result?
27. Positive
28. Negative
29. Indeterminate
30. Did not receive my results
31. In the **past 6 months**, have you been tested for sexually transmitted infections (STIs), including syphilis, gonorrhea, chlamydia, herpes genital warts, or hepatitis B or C? *
32. Yes
33. No (Logic: Jump to D1)
34. Please indicate whether you have been diagnosed with any of the following sexually transmitted infections in the **past 6 months**? Check all that apply. *
35. Chlamydia
36. Genital warts, anal warts, HPV
37. Gonorrhea
38. Hepatitis B
39. Hepatitis C
40. Herpes, HSV1/HSV2
41. Syphilis
42. None of the above
43. **PrEP Knowledge/Attitudes**
44. Before today, had you ever heard of PrEP? *
45. Yes
46. No (Logic: Jump to D3)
47. Where did you hear about PrEP? Check all that apply. *
48. A friend or family member
49. A sex partner
50. At a community meeting
51. An HIV prevention counselor
52. On a website
53. From a poster or flyer
54. Blued app
55. Other, specify ______
56. How effective is PrEP at preventing HIV infection if a person takes their pills every day? *

Not effective --------------------------------•------------------------------Very effective

(0%) (100%)

---Page break---

# **PrEP Intention and use**

**Pre-exposure prophylaxis (PrEP) is taking an antiretroviral pill, every day to reduce a person’s chance of getting HIV. Research has shown PrEP is over 99% effective in preventing HIV when taken daily. Currently in China, to get PrEP a person needs to go to the doctor, get tested for HIV every three months, and pay for the medication. This approach is approved by the China’s NMPA and US FDA, with quality clinical trial evidence supporting the strategy as an effective way to prevent HIV.**

1. If it were available to you for a cost of CNY 500/month, how likely would you be to take PrEP in the next 6 months? * [Stage 2a: Contemplation]
2. I would definitely take it
3. I would probably take it
4. I would probably not take it
5. I would definitely not take it
6. If it were available to you for free, how likely would you be to take PrEP in the next 6 months? * [Stage 2a: Contemplation]
7. I would definitely take it
8. I would probably take it
9. I would probably not take it
10. I would definitely not take it
11. Do you believe that you are currently an appropriate candidate for PrEP? * [Stage 2b: Contemplation]
12. Yes, I am definitely an appropriate candidate
13. I am probably an appropriate candidate
14. I am probably not an appropriate candidate
15. No, I am definitely not an appropriate candidate
16. If you were interested in getting PrEP, do you have or know where to access PrEP? * [Stage 3a: Preparation]
17. Yes
18. No
19. Do you plan to begin PrEP in the next month? * [Stage 3b: Preparation]
20. I will definitely begin taking PrEP
21. I will probably begin taking PrEP
22. I probably will not begin taking PrEP
23. I definitely will not begin taking PrEP
24. Have you ever spoken to a medical provider about starting PrEP? * [Stage 4a: Action/initiation]
25. Yes
26. No
27. Have you ever taken PrEP? *
28. Yes
29. No (Logic: Jump to F1)
30. In the past 3 months, have you used PrEP?
31. Yes
32. No (Logic: Jump to F1)
33. Are you currently taking PrEP? [Stage 4: Action/initiation]
34. Yes
35. No (Logic: Jump to F1)
36. In the last seven days, how many days did you miss a dose of PrEP? * [Stage 5: Adherence]

---Page break---

# **PrEP formulation preferences**

1. If *daily oral* PrEP were available from your local clinic and you could access it for free, would you go to your doctor in the next month to start *daily oral* PrEP? *
2. No
3. Yes
4. Don’t know

There is another way that some people take PrEP pills called *on-demand oral* PrEP. With on-demand oral PrEP you take a series of pills around the time when you have sex. This means taking 2 pills 2-24 hours before sex, 1 pill 24 hours after the first dose, and 1 pill 24 hours after the second dose (dosing sometimes called 2-1-1). Some studies found that on-demand oral PrEP is just as effective at preventing HIV infection as daily oral PrEP. This approach is not currently approved by the China’s NMPA or US FDA, but has quality clinical trial evidence supporting the strategy as an effective way to prevent HIV.

1. Before this survey, have you heard of *on-demand oral* PrEP? *
2. No
3. Yes
4. Don’t know
5. If *on-demand oral* PrEP were available from your local clinic and you could access it for free, would you go to your doctor in the next month to start *on-demand oral* PrEP? *
6. No
7. Yes
8. Don’t know

---Page break---

An *injectable form of PrEP* given in your buttocks has been found to be just as effective as daily oral PrEP in preventing HIV infection. You would have to see a doctor to start injectable PrEP and continue to see them every 2 months to stay on it. The possible side effects of injectable PrEP are mild-to-moderate pain at the injection site that lasts 2 to 7 days and mild rash at the injection. This approach is not currently approved by the China’s NMPA, but is approved by US FDA with quality clinical trial evidence supporting the strategy as an effective way to prevent HIV.

1. Before this survey, have you heard of *injectable* PrEP? *
2. No
3. Yes
4. Don’t know
5. If *injectable* PrEP were available from your local clinic and you could access it for free, would you go to your doctor in the next month to start *injectable* PrEP? *
6. No
7. Yes
8. Don’t know
9. You said you would be willing to start these ways of taking PrEP. If the same doctor offered all these options to you, rank them in order of which you would be most likely to choose. Please enter the number in the [ ]. [1 is the one you would be most likely to choose, and 3 is the least likely to choose.] *[if F1=a and F3=a]

[ ] Daily PrEP Pills

[ ] On-demand PrEP Pills

1. You said you would be willing to start these ways of taking PrEP. If the same doctor offered all these options to you, rank them in order of which you would be most likely to choose. Please enter the number in the [ ]. [1 is the one you would be most likely to choose, and 3 is the least likely to choose.] * [if F1=a and F5=a]

[ ] Daily PrEP Pills

[ ] Injectable PrEP

1. You said you would be willing to start these ways of taking PrEP. If the same doctor offered all these options to you, rank them in order of which you would be most likely to choose. Please enter the number in the [ ]. [1 is the one you would be most likely to choose, and 3 is the least likely to choose.] * [if F3=a and F5=a]

[ ] On-demand PrEP Pills

[ ] Injectable PrEP

1. You said you would be willing to start these ways of taking PrEP. If the same doctor offered all these options to you, rank them in order of which you would be most likely to choose. Please enter the number in the [ ]. [1 is the one you would be most likely to choose, and 3 is the least likely to choose.] * [if F1=a and F3=a and F5=a]

[ ] Daily PrEP Pills

[ ] On-demand PrEP Pills

[ ] Injectable PrEP

---Page break---

1. Which of the following describe the reason(s) why you do not want to start on any forms of PrEP for free? * [check all that apply] (logic: if F1=b or c, and F3= b or c, and F5 = b or c)
2. I am concerned about the side-effects
3. I could not follow the PrEP prescriptions strictly to obtain effective protection
4. I am in a monogamous relationship with an HIV-negative partner
5. I am not sexually active
6. I prefer to use other methods to protect myself from HIV
7. I worry that people will think that I have HIV when they see me taking the pill
8. I worry that people will think that I am very sexually active because I am on PrEP
9. I worry my partner will not support me to take PrEP
10. I worry someone who live with me will see me taking PrEP
11. I am not able to start PrEP due to Covid impact
12. Please rank your reason(s) of not starting PrEP from most concerned to least concerned.

[ ] (options coming from F7)

[ ]

--- page break--

# **HIV Knowledge**

G1. Please answer the following statements to the best of your ability. *

|  | Yes | No | I don't know |
| --- | --- | --- | --- |
| Do you know anyone who has HIV or AIDS? |  |  |  |
| A person who has HIV can look healthy. |  |  |  |
| There is a vaccine that can stop you from getting HIV. |  |  |  |
| The risk for getting HIV is very low for deep kissing (tongue in partner’s mouth) even if your partner has HIV. |  |  |  |
| Nearly all HIV transmission comes from having lots of boyfriends or hook-ups. |  |  |  |
| The risk for getting HIV is very low when having oral sex. |  |  |  |
| A person is more likely to get HIV from receptive sex (bottom) than insertive sex (top). |  |  |  |
| Showering or washing your genitals/private parts after having sex will make you less likely to get HIV. |  |  |  |

---Page break---

# **PrEP Stigma**

**As a reminder, Pre-exposure prophylaxis (or PrEP) is when people at risk for HIV take daily antiviral medicine to prevent HIV.**

H1. If you were to take PrEP, please indicate how much you agree with the following statements: *

|  | **Strongly Disagree**  **(1)** | **Disagree**  **(2)** | **Neutral**  **(3)** | **Agree**  **(4)** | **Strongly Agree**  **(5)** |
| --- | --- | --- | --- | --- | --- |
| I would feel ashamed to take PrEP pills in front of others. |  |  |  |  |  |
| Someone taking PrEP should keep their pills hidden. |  |  |  |  |  |
| People experience negative judgment because they take PrEP. |  |  |  |  |  |
| I would have sex with someone who is taking PrEP. |  |  |  |  |  |
| Someone taking PrEP would be seen by others as slutty. |  |  |  |  |  |
| People taking PrEP receive praise for being responsible. |  |  |  |  |  |
| My friends would be supportive of me taking PrEP. |  |  |  |  |  |
| People experience problems when they tell their sex partner(s) they are taking PrEP. |  |  |  |  |  |
| I would feel proud to take PrEP every day. |  |  |  |  |  |
| People taking PrEP experience verbal harassment. |  |  |  |  |  |
| People on PrEP are taking care of their health. |  |  |  |  |  |
| My *family* would be supportive of me taking PrEP. |  |  |  |  |  |

---Page break---

1. **Mental Health**

**I1. Over the last two weeks**, how often have you been bothered by any of the following problems? *

|  | **Not at all**  **(0)** | **Several days**  **(1)** | **More than half the days**  **(2)** | **Nearly every day**  **(3)** | **Decline to answer** |
| --- | --- | --- | --- | --- | --- |
| Little interest or pleasure in doing things |  |  |  |  |  |
| Feeling down, depressed or hopeless |  |  |  |  |  |
| Feeling nervous, anxious, or on edge |  |  |  |  |  |
| Not being able to stop or control worrying |  |  |  |  |  |

---Page break---

1. **Order History**

J1. In the past 3 months, which of the following services or products have you ever ordered through the Blued platform? Check all that apply. *

1. Condoms
2. Lubricant
3. HIV self-testing kits
4. PrEP

J2. Have you used the condom(s) you ordered through Blued? * (if J1=a)

1. Yes, used all
2. Yes, used some
3. No at all

J3. Have you used the lubricant(s) you ordered through Blued? * (if J1=b)

1. Yes, used all
2. Yes, used some
3. No at all

J4. Have you used the HIV self-testing kit(s) you ordered through Blued? * (if J1=c)

1. Yes, used all
2. Yes, used some
3. No at all

J5. Have you taken the PrEP drugs you ordered through Blued? * (if J1=d)

1. Yes, taken all
2. Yes, taken a part
3. No at all

---Page break---

**Enrollment information for follow-up and compensation purpose**

**Remind again:**

Fill out this questionnaire and follow-up questionnaires every 3 months, and each time you will get a Jingdong card worth 100 yuan as survey compensation. If you complete all 6 questionnaires, you will receive an additional 400 yuan in compensation. Up to a total of 1,000 yuan in compensation can be obtained.

Project research subjects will also receive free PrEP drugs, condoms, lubricants and testing reagents during the project.

If you are willing to participate in this research project, please leave your contact information so that we can issue investigation compensation and follow-up contact.

First name: ____________ Last Name: ________________

Primary phone number: ___________________________

Second phone number (optional): ___________________

Email address: ________________________

WeChat id #1: ________________________

WeChat id #2 (optional): ________________

Preferred method of contact: (check all that apply):

1. Primary phone number
2. Second phone number
3. Email
4. WeChat 1
5. WeChat 2
