# Supplementary material for: Integrating a Combination HIV Prevention Intervention Into a Widely Used Geosocial App for Chinese Men Who Have Sex With Men: Protocol for a Single-Arm Pilot and Repeated Cross-Sectional Study
Source: JMIR Res Protoc. 2025 Sep 29;14:e69536. doi: 10.2196/69536 (PMC12519034; doi:10.2196/69536)
Supplement: Multimedia Appendix 6 [file resprot_v14i1e69536_app6.pdf]

SUMMARY STATEMENT
(Privileged Communication)

PROGRAM CONTACT:
Cherlynn Mathias
240-292-4791
cmathias@niaid.nih.gov

Release Date: 08/17/2018
Revised Date:

Application Number: 1 R01 AI143875-01

Principal Investigators (Listed Alphabetically):

SIEGLER, AARON JULIUS (Contact)
SULLIVAN, PATRICK SEAN

Applicant Organization: EMORY UNIVERSITY

Review Group: ZRG1 AARR-K (03)
Center for Scientific Review Special Emphasis Panel
Member Conflict: AIDS and Related Research
AIDS - EXP. REV.

Meeting Date: 08/08/2018
Council: OCT 2018
Requested Start: 12/01/2018
RFA/PA: PAR16-124
PCC: A22
Dual IC(s): MH

Project Title: Integrating a combination HIV prevention intervention into a widely-used geosocial app for Chinese MSM
SRG Action: Impact Score:29 Percentile:13 #
Next Steps: Visit https://grants.nih.gov/grants/next_steps.htm
Human Subjects: 30-Human subjects involved - Certified, no SRG concerns
Animal Subjects: 10-No live vertebrate animals involved for competing appl.
Gender: 3A-Only men, scientifically acceptable
Minority: 5A-Only foreign subjects, scientifically acceptable
Children: 3A-No children included, scientifically acceptable

[redacted]

ADMINISTRATIVE BUDGET NOTE: The budget shown is the requested budget and has not been adjusted to reflect any recommendations made by reviewers. If an award is planned, the costs will be calculated by Institute grants management staff based on the recommendations outlined below in the COMMITTEE BUDGET RECOMMENDATIONS section.

1R01AI143875-01 SIEGLER, AARON

RESUME AND SUMMARY OF DISCUSSION: This application seeks support to develop, test and evaluate a plug-in modification of a geosocial networking app (BlueD) that is widely used in China among men who have sex with men (MSM) to find partners; the plug-in would provide information on prevention and linkage to home-based screening and care. The application addresses the important problem of effective interventions with MSM in China, the only risk group in China with increasing rates of HIV detection; adaptation of an already widely used app is especially attractive. The project involves a review of the literature, development of a mathematical model, followed by the intervention. There is some concern that the literature review does not include the Chinese literature, especially the failure to include a Chinese national database that could inform the program. The investigative team is impressive and has the necessary scientific expertise, but there is some concern that the US-based researchers have no Chinese cultural or language expertise and the Chinese investigator recruited for the program lacks expertise in the socio-cultural factors involved in HIV prevention. An important strength is the formal plan for collaboration with BlueD to develop the plug-in. The adaptation of an existing app is highly innovative, especially since app usage is high among the target population; it is noted that this project “is trying to get ahead of the curve”. Additionally, the model is likely to translate to other countries. Some reviewers see a significant weakness in failing to address social/cultural issues that are specific to Chinese MSM. For example, most MSM in China are married and there is very low use of pre-exposure prophylactic (PrEP) therapy, in part because of the stigma involved and because of the need to register. Some reviewers emphasized concerns about political considerations in China that could affect implementation. Although views remain disparate at the end of discussion, most are highly supportive of this novel proof of concept project that has the potential to change the field.

DESCRIPTION (provided by applicant): A number of HIV prevention interventions are known to be effective: HIV testing and linkage to care as appropriate, condom use, and HIV pre-exposure prophylaxis (PrEP). Unfortunately, achieving sufficient use of these prevention services has been challenging. In China, men who have sex with men (MSM) are the only risk group with increasing HIV case detection, representing 26% of new cases diagnosed in 2014. One way to obtain increased prevention intervention use would be to help individuals understand when they should be using certain prevention services, and to make accessing services less burdensome. The proposed research will involve conducting a literature review and developing mathematical models to explore different scenarios of providing a combination HIV prevention service intervention for MSM in China. Using information from this exercise, we will develop and pilot test a combination HIV intervention built into a smartphone app. The intervention will include home care services such as home HIV self-testing and home ordering of prevention products such as condoms. If the intervention proves to be feasible and acceptable, the pilot test will facilitate design of a larger study of intervention efficacy. The intervention will be built into an existing, widely-accessed geosocial networking app used by MSM. By building the intervention into a platform already used by millions of individuals, we anticipate that, in the event that the intervention is demonstrated to be efficacious, scale-up of the intervention will be readily achievable. The proposed research seeks to develop mathematical models and an intervention that would reduce the incidence of HIV/AIDS, meeting an overarching research priority identified in the Strategic Plan of the NIH Office of AIDS Research.

PUBLIC HEALTH RELEVANCE: This study seeks to conduct a literature review and develop models that simulate an HIV epidemic. We will use lessons from this process to work with an app company with a large user-base to develop and test an intervention that would include home-based services. The proposed research will allow us to understand the acceptability and feasibility of the prevention intervention.

CRITIQUE 1:

Significance: 6
Investigator(s): 6
Innovation: 5
Approach: 7
Environment: 1

Overall Impact: The population of men who have sex with men (MSM) in China continues to witness an increase in HIV diagnosis. The proposed research aims to employ a combination of a literature review and mathematical modeling to gauge the epidemic. Lessons learned from these will guide the development of an app-based HIV prevention/intervention package to "… test an intervention that would include home-based services. …. to understand the acceptability and feasibility of the prevention intervention." The study primarily focuses on behavioral approaches (e.g., home testing; PrEP uptake); it fails to address important issues such as discrimination and stigma in the literature, which are the root causes for the high HIV incidence and prevalence in this population which are reflected in low testing practices, among other indicators. For example, home testing may offer privacy but it does not directly address discrimination and/or stigma, in that should a MSM test positive and want treatment he still needs to interact with the treatment-and-care system which are "centrally operated" by the government. Moreover, there is no formally organized or national PrEP program, thus it is not realistic to expect individuals to pay out of their own pockets in the "long haul." In short, the practicality or sustainability ("public health significance") of such a package is debatable at best. The literature review (e.g., PrEP in China; socio-cultural factors such as discrimination and stigma) is inadequate. The study states that it is not a clinical trial. Nonetheless, participants will be assigned to an "intervention," though the study will not evaluate the effect of the intervention on the participants nor evaluate a health-related biomedical or behavioral outcome.

1. Significance:

Strengths

- MSM continue to bear the brunt of the HIV epidemic in China -- sadly this is on par with countries such as Thailand.

Weaknesses

- The emphasis on behaviors without addressing their motivations are misguided.
- The literature on HIV prevention targeting MSM in China consistently argue that issues such as discrimination, stigma, homophobia are the main drivers of the epidemic. For example, a significant of the MSM population in China are married and/or engaged in bisexual behaviors and/or relationships with women. A married MSM who wants to take PrEP is going to raise suspicions in his wife. The failure to address these issues are missed opportunities.
- The entire premise of the application is rather American centric, that is, there is a lack of an appreciation of cultural contexts and practices.

2. Investigator(s):

Strengths

- The U.S.-based team is impressive and has the technical expertise to achieve the proposed specific aims.

- The inclusion of Dr. Vermund as a consultant who had a MP3 targeting MSM in China is a strength.

Weaknesses

- None of the U.S.-based researchers has any cultural and/or language expertise of the country.
- Dr. Guodong Mi, an in-country investigator who is trained in internal medicine and epidemiology, does not have the scientific expertise to address the influences of these socio-cultural factors in HIV prevention.

3. Innovation:

Strengths

- Integrate an app-based approach in a popular social media platform targeting gay and bisexual men in China.

Weaknesses

- It is questionable if the use of smartphone with an app can be realistically implemented in China. This application does not provide any preliminary data specific to the cultural and in-country contexts. This is not a scientific issue but a “political” issue, and this application does not provide enough assurance this can be achieved.

4. Approach:

Strengths

- A track record in methodologies for conducting a similar study.

Weaknesses

- The framework of the literature search is biased in favor of English-language publications. There is a national database in Chinese but it is not mentioned in the application.
- Failure to address socio-cultural factors such as stigma which are one of the root causes of the HIV epidemic among MSM in China.
- Recently, China’s NCAIDS has conducted a large-scale study on PrEP targeting MSM (Chengdu was one of the sites with the same NGO as in the proposed study). This is not mentioned in the application nor its published findings. In fact, PrEP use (free of charge) is less than 3%.
- Without preliminary data regarding the use of smartphone with an app specifically addressing HIV prevention targeting MSM in China, the feasibility of the study is questionable amidst the political climate in the country.
- PrEP medication will be donated by Gilead, but uptake is a big unknown. The national study has a sample size of over 1,000 and less than 3% used PrEP. In fact, some quit midway through the study.
- What happens when a MSM (“closeted” and “married”) is tested HIV-positive, what are the safeguard measures? If he is tested positive legally he needs to be “registered” with the national system (“CRIMS”) in order to be eligible for free treatment and care. There is no description on some of these contingencies.

5. Environment:

Strengths

- Seem appropriate

Weaknesses

- None noted

Protections for Human Subjects:

Unacceptable Risks and/or Inadequate Protections

- MSM who tested positive using home self-testing kits may not want to disclose their HIV status to others (e.g., sexual partners, significant others). There is no provision on how to address these scenarios.

Inclusion of Women, Minorities and Children:

- Sex/Gender: Distribution justified scientifically.
- Race/Ethnicity: Distribution justified scientifically.
- Inclusion/Exclusion of Children under 18: Excluding ages <18; justified scientifically.

Vertebrate Animals:

Not Applicable (No Vertebrate Animals)

Biohazards:

Not Applicable (No Biohazards)

Resource Sharing Plans:

Acceptable

Authentication of Key Biological and/or Chemical Resources:

Not Applicable (No Relevant Resources)

Budget and Period of Support:

Recommend as Requested

CRITIQUE 2:

Significance: 2
Investigator(s): 1
Innovation: 3
Approach: 3
Environment: 1

Overall Impact: This four-year R01 aims to conduct an umbrella review of literature, mathematical model and then to conduct a pilot intervention (n=400) using the Blued (Blued+) platform to focus on combination HIV prevention services for MSM in China, totaling almost 8,500 participants. The integration of HIV prevention services in a geosocial networking app can have a tremendous public health impact and can serve as a guide for other geosocial apps – Grindr, Jack'd, etc. Enthusiasm for the proposed project is high and this project can truly impact the field.

1. Significance:

Strengths

- The scientific premise for the proposed study is very strong--HIV is a public health priority in China, especially among MSM.
- Geosocial networking apps are highly popular among MSM and Blued is the largest of such apps for MSM in China. Integration of HIV prevention services into an existing app (as opposed to creating a new HIV health app) likely will result in increased uptake of the HIV services offered and can potentially have a tremendous public health impact because app use may be in a high-risk time period.
- Strong pilot data demonstrate that the intervention is needed and may be effective; e.g., >60% of MSM who had never received an HIV test indicated they would order a home-HIV test if available through Blued.

Weaknesses

- None noted

2. Investigator(s):

Strengths

- Dr. Siegler has prior experience and expertise in HIV prevention and technology in MSM; Dr. Sullivan is a very strong HIV epidemiologist with significant experience in MSM research and technology.
- Formal collaboration with BlueD is important.
- Investigators have previous experience with the MP3 mechanism, including co-Investigator Dr. Vermund who (among others) also has significant research experience in China.
- This truly represents the best team to conduct the proposed research, with complementary (not duplicative) expertise.

Weaknesses

- The Multiple Principal Investigator plan is lacking detail on what each Principal Investigator will be responsible for--it is very general.

3. Innovation:

Strengths

- Testing an HIV intervention via a geosocial app (BlueD) is innovative; no HIV prevention apps developed by NIH-funded researchers are freely available to and used by the general public, including home HIV testing, condoms, referral to PrEP services and referral to STI testing.

Weaknesses

- None noted

4. Approach:

Strengths

- The scientific rigor and transparency is very strong—described below.
- The literature reviews will include examining the grey literature.
- The mathematical models will be informed by large cross-sectional surveys (2,000 per year).
- Qualitative research will inform the intervention, including cognitive interviews. The theory-based intervention (potentially to include HIV testing, condom use, PrEP use, etc.) will be conducted in 2 cities (n=400), which increases the generalizability. Participants in the intervention will be randomly selected from BlueD app users. The intervention will utilize an interrupted time series design, which is strong. In addition, there are strong intervention acceptability and feasibility outcomes, including objective measures of PrEP.
- There is a plan to develop an efficacy intervention, following the pilot intervention.
- Sex as a biological variable: Focus on MSM is scientifically justified.

Weaknesses

- No age restriction; just 18 and over, which may reduce intervention salience and therefore efficacy.
- $1 incentive for the 8,000 participants who take the cross-sectional survey does not seem feasible or acceptable.

5. Environment:

Strengths

- The proposed sites for the work (Emory, University of North Carolina, Danlan, etc.) are great.

Weaknesses

- None noted

Protections for Human Subjects:

Acceptable Risks and/or Adequate Protections

Inclusion of Women, Minorities and Children:

- Sex/Gender: Distribution justified scientifically.
- Race/Ethnicity: Distribution justified scientifically.
- Inclusion/Exclusion of Children under 18: Excluding ages <18; justified scientifically.

Vertebrate Animals:

Not Applicable (No Vertebrate Animals)

Biohazards:

Not Applicable (No Biohazards)

Resource Sharing Plans:

Acceptable

Authentication of Key Biological and/or Chemical Resources:

Not Applicable (No Relevant Resources)

Budget and Period of Support:

Recommend as Requested

CRITIQUE 3:

Significance: 1
Investigator(s): 1
Innovation: 1
Approach: 1
Environment: 1

Overall Impact: This demonstration "pilot" study to develop, test and evaluate a plug-in modification of a widely used social networking app (BlueD) among MSM in China to provide prevention knowledge, linkage to care, screening for STI and PrEP tool is urgently needed. This work will be conducted by a team of expert investigators who have conducted pilot feasibility and acceptance studies of app technology among MSM in China and South Africa. As such expectation of the successful completion of this work is very high. It is innovative with high impact. It was a great pleasure to read this well written application.

1. Significance:

Strengths

- This is a novel technology expansion of a widely used social networking app (BlueD) used Chinese MSM (estimated 12 million users each month) to provide directed pathways for both in-clinic and at home HIV testing and potential PrEP delivery (with the expectation of Chinese FDA approval of PrEP within the grant period).
- Development of the BlueD (and projected Blued+) app is a US-China cooperative project.
- The HIV prevalence among MSM in Chinese urban centers is 4.9%, but survey data collected by these investigators have shown very low HIV testing, very high condomless sexual acts, and low knowledge of PrEP and very low current PrEP use. For all these reasons, a novel, acceptable technology-drive HIV prevention knowledge and linkage to care tool is urgently needed.
- The culling of relevant information informing scientists and community groups of what an acceptable technology tool would look like and how it would function through the systematic literature review of HIV prevention strategies along with mathematical modeling and evaluation for implementation using study outcomes (Figure 5) is very exciting.

Weaknesses

- None

2. Investigator(s):

Strengths

- All of the scientific investigators (prevention, modeling, participant outreach, and technology development) in the US and China are highly qualified. This is a stellar team.

Weaknesses

- The budgetary justification for the Principal Investigators (Drs. Siegler and Sullivan) is written very similarly regarding responsibilities; more delineation is necessary.

3. Innovation:

Strengths

- The development of an easy, portable app to deliver HIV testing opportunities, condoms, and PrEP among millions of Chinese MSM who currently use the app for social networking reminds me of how my Amazon App helps me order food that is delivered to my door. This type of enterprise is what is expected and resonates with younger MSM, who like in the US, are at the highest risk of HIV infection and have the highest need for prevention methods.

Weaknesses

- None noted

4. Approach:

Strengths

- The methodical approach of literature searching, modeling, feedback from community, and development and testing of BlueD+ iteratively in this demonstration project is extremely well written and justified.

Weaknesses

- Investigators were very forthright about stating the limitations of this project (particularly that the scope of this demonstration project will not be available to all MSM and that it is a feasibility, not an efficacy study).

5. Environment:

Strengths

- All facilities in the application are very satisfactory for a successful completion of this study.

Weaknesses

- Conducting studies between US and China can be logistically challenging vis a vis timely digital communication.

Protections for Human Subjects:

Acceptable Risks and/or Adequate Protections

Inclusion of Women, Minorities and Children:

- Sex/Gender: Distribution justified scientifically.
- Race/Ethnicity: Distribution justified scientifically.
- Inclusion/Exclusion of Children under 18: Excluding ages <18; justified scientifically.

Vertebrate Animals:
Not Applicable (No Vertebrate Animals)

Biohazards:
Not Applicable (No Biohazards)

Resource Sharing Plans:
Acceptable

Authentication of Key Biological and/or Chemical Resources:
Not Applicable (No Relevant Resources)

Budget and Period of Support:
Recommend as Requested

THE FOLLOWING SECTIONS WERE PREPARED BY THE SCIENTIFIC REVIEW OFFICER TO SUMMARIZE THE OUTCOME OF DISCUSSIONS OF THE REVIEW COMMITTEE, OR REVIEWERS' WRITTEN CRITIQUES, ON THE FOLLOWING ISSUES:

PROTECTION OF HUMAN SUBJECTS: ACCEPTABLE
Although there was some discussion about the protection of human subjects, the committee decided that risks and protections were adequately described and the protection of human subjects was acceptable.

INCLUSION OF WOMEN PLAN: ACCEPTABLE

INCLUSION OF MINORITIES PLAN: ACCEPTABLE

INCLUSION OF CHILDREN PLAN: ACCEPTABLE

COMMITTEE BUDGET RECOMMENDATIONS: The budget was recommended as requested.

Footnotes for 1 R01 AI143875-01; PI Name: Siegler, Aaron Julius

Ad hoc or special section application percentiled against "Total CSR" base.

NIH has modified its policy regarding the receipt of resubmissions (amended applications). See Guide Notice NOT-OD-14-074 at http://grants.nih.gov/grants/guide/notice-files/NOT-OD-14-074.html. The impact/priority score is calculated after discussion of an application by averaging the overall scores (1-9) given by all voting reviewers on the committee and multiplying by 10. The criterion scores are submitted prior to the meeting by the individual reviewers assigned to an application, and are not discussed specifically at the review meeting or calculated into the overall impact score. Some applications also receive a percentile ranking. For details on the review process, see http://grants.nih.gov/grants/peer_review_process.htm#scoring.

MEETING ROSTER

The roster for this review meeting is displayed as an aggregated roster that includes reviewers from multiple CSR Special Emphasis Panels of the AIDS and Related Research Integrated Review Group for the 2018/10 council round.
This roster for CSR is available at:
http://public.era.nih.gov/pubroster/Reports?DOCTYPE=SEP&DESFORMAT=PDF&AGENDA_SEQ_NUM_P=350027
